# Supplementary material for: Genome-wide approaches for the identification of markers and genes associated with sugarcane yellow leaf virus resistance
Source: Sci Rep. 2021 Aug 3;11:15730. doi: 10.1038/s41598-021-95116-1 (PMC8333424; doi:10.1038/s41598-021-95116-1)
Supplement: Supplementary file 1 — Supplementary Information. [file 41598_2021_95116_MOESM1_ESM.pdf]

Genome-wide approaches for the identification of markers and genes associated with sugarcane yellow leaf virus resistance

Ricardo José Gonzaga Pimenta, Alexandre Hild Aono, Roberto Carlos Villavicencio Burbano, Alisson Esdras Coutinho, Carla Cristina da Silva, Ivan Antônio dos Anjos, Dilermando Perecin, Marcos Guimarães de Andrade Landell, Marcos Cesar Gonçalves, Luciana Rossini Pinto, Anete Pereira de Souza

Supplementary Material

Supplementary Results

1. Phenotypic data analyses

97 sugarcane accessions inoculated with SCYLV were evaluated for the severity of SCYL symptoms and for viral titer estimated by relative and absolute RT-qPCR quantification in two consecutive years. We observed a skew in the distribution of symptom severity data towards the absence of symptoms, and despite normalization procedures, these data did not follow a normal distribution (p = 2.2e-16). This was not the case for SCYLV titer; both the relative (p = 0.999) and absolute (p = 0.930) quantification data were successfully normalized. Plots of the distribution of the best linear unbiased predictors (BLUPs) calculated for the three traits analyzed and of the correlations between them are depicted in Supplementary Fig. 2.

2. Genotyping

Genotyping the 97 accessions of the panel using 12 SSRs and 35 AFLPs generated 981 polymorphic markers (167 SRR fragments and 814 AFLP fragments). Upon filtering, four genotypes (74, 93, 96 and 97) and 319 markers were removed as they presented call rates below 90% or minor allele frequency (MAF) below 10%. Therefore, 93 genotypes and 662 dominant markers were retained for further analyses that involved them.

The sequencing of the GBS library including 94 sugarcane genotypes generated 863,889,004 raw reads, 87.88% of which presented a Q-value above 30. Genotypes 31 and 77 presented very low amounts of generated data (0.19 and 1.5 KB, respectively) in contrast with the library average (598 KB) and were therefore excluded from subsequent analyses to avoid biasing. The results from tag mapping against each genomic reference using BWA and Bowtie2 aligners can be found in Supplementary Table 6. To avoid sampling of duplicated regions – which are abundant in the sugarcane genome and could bias the calculation of allele proportions (APs) – we did not include tags with multiple alignments in the ensuing analyses. BWA presented slightly superior results in terms of uniquely

mapped tags and was therefore chosen as the aligning tool. Regarding the nine genomic references tested for variant calling, the methyl-filtered genome of the cultivar SP70-1143 resulted in the largest number of isolated markers among all references, yielding 60,173 SNPs and 43,054 indels after rigorous filtering (Supplementary Table 7). However, knowing that a high-quality genome assembly can greatly improve the results of GWASs and allow the estimation of long-distance LD, we opted to perform ensuing analyses with markers obtained from a reference that not only yielded a large number of markers but also provided reliable information on their position. Thus, we chose to use the monoploid chromosome set isolated from the *S. spontaneum* genome as a reference; despite not yielding the highest number of markers (38,710 SNPs and 32,178 indels), this genome had a performance superior to most of the other references tested here and, unlike many others, provided markers with information of position at chromosome level.

3. Genetic structure analyses

The genetic structure of the panel was investigated using two separate sets of markers – AFLPs and SSRs scored as dominant and codominant SNPs and indels with AP information – and three different approaches – a discriminant analysis on principal components (DAPC), a principal component analysis (PCA) followed by k-means and a Bayesian clustering implemented in STRUCTURE software. These three methods were first performed with dominant markers. The DAPC was performed retaining 70 principal components (PCs) that together explained ~90% of the observed variance (Supplementary Fig. 7a); the Bayesian information criterion (BIC) curve indicated k = 3 as the most likely number of clusters in the panel (Supplementary Fig. 7b). Therefore, genotypes were separated into three clusters (A1-3); the plotting of the panel elements onto the first two linear discriminants from this analysis is shown in Supplementary Fig. 10a. Despite resulting in the subdivision of the panel into four clusters (B1-4), as indicated by two out of three statistical methods performed to assess the optimal number of clusters in the panel (Supplementary Fig. 8a-c), clustering by PCA followed by k-means yielded results that shared similarities with the DAPC. Genotypes forming B1 and B4, for instance, were entirely represented by A3 and A1, respectively. Supplementary Fig. 10b depicts a projection of groups identified by k-means onto the PCA's first two PCs, which accounted for ~18% of the variation in the genotypic data. Last, based on Bayesian clustering, both ΔK and LnP(D) methods clearly indicated K = 2 as the most likely number of clusters in the panel (Supplementary Fig. 9a and b, respectively). Therefore, we present here a plot of the estimated association coefficients of individuals for these two clusters (Supplementary Fig. 10c), named C1 and C2. Clustering by this method was highly consistent with

clustering by k-means, as C1 corresponded to the union of B1 and B2, and C2 corresponded to the union of B3 and B4.

These same three analyses were next performed with codominant markers. DAPC was performed retaining 60 PCs explaining ~90% of the observed variance (Supplementary Fig. 11a); the BIC curve indicated k = 3 as the most likely number of clusters in the panel (Supplementary Fig. 11b). Thus, the panel was separated into three groups (D1-3); the plotting of the panel elements onto the first two linear discriminants from this analysis is shown in Supplementary Fig. 14a. PCA followed by k-means was executed assuming the existence of two clusters (E1 and E2), as clearly indicated by all three testing methods employed (Supplementary Fig. 12a-c). Despite indicating the subdivision of the panel into two clusters, the attribution of genotypes to these groups shared similarities with the DAPC clustering. E1 corresponded to D2, and E2 was equivalent to D1 and D3 combined. Supplementary Fig. 14b depicts a projection of these groups onto the PCA's first two PCs, which accounted for ~33% of the variation in the genotypic data. Finally, based on Bayesian clustering, both ΔK and LnP(D) methods indicated K = 2 as the most likely number of clusters in the panel (Supplementary Fig. 13a and b, respectively). Thus, we present here a plot of the estimated association coefficients of individuals for these two clusters, named F1 and F2 (Supplementary Fig. 14c). Clustering by this procedure was also consistent with clustering by the other two methods, as F1 corresponded to the union of D2 and D3, and F2 corresponded to D1.

Supplementary Tables

Supplementary Table 1. List of genotypes in the association panel with their respective parents.

ID Number	Genotype	Female parent	Male Parent
1	US571415	*S. spontaneum*	SP701143
2	Cana alho	*S. officinarum*	Unknown
3	IACSP012410	R570	IACSP933046
4	IACSP046007	PO8862	IAC912195
5	IACSP046059	IACSP953018	SP775181
6	IACSP953028	IACSP953028	Unknown
7	NG5712	*S. robustum*	Unknown
8	IACSP042516	IAC912288	CT961414
9	Krakatau	*S. spontaneum*	Unknown
10	IN8482	*S. spontaneum*	SP823530
11	IACSP042508	SP913059	Unknown
12	IACSP955037	SP842066	IACSP973391
13	IACCTC059630	IACSP972053	SP80185
14	IACSP046053	IACSP956114	IAC873396
15	IACSP993009	CP701133	Unknown
16	IS76155	*S. officinarum*	RB855113
17	IACSP012421	RB835486	SP847017
18	IACSP042521	IAC912288	SP88721
19	IACSP046058	IACSP953018	SP87365
20	IACSP991020	SP851682	Unknown
21	IACSP045065	IACSP956114	RB72454
22	IACCTC069767	IACSP963069	SP803280
23	IACSP985046	SP813251	Unknown
24	IACSP018034	IAC913093	Unknown
25	IACSP993357	SP921584	Unknown
26	IACCTC059578	Whitetransparent	SP963092
27	IACCTC055580	CTC4	IACSP933046
28	IACSP976628	RB835486	SP931124
29	IACSP976680	SP842066	Unknown
30	IACCTC059534	IACSP953028	Unknown
31	IACSP972084	RB855035	SP775181
32	CTC15	SP842025	Unknown
33	IAC914168	SP813137	IAC873396
34	IACBIO232	RB855465	CTC9
35	IACBIO241	RB855465	SP963092
36	IACBIO257	NG26011	IAC863154
37	IACBIO266	NG26011	RB855156
38	IACBIO270	SES069	Unknown
39	IACBIO271	SES069	SP80185

40	IACBIO273	SES069	Unknown
41	IACBIO275	SES069	Unknown
42	IACBIO277	SES069	IACSP972077
43	IACBIO279	SES069	IAC873396
44	IACCTC053616	IAC915155	RB85453
45	IACCTC056518	SP911049	SP832847
46	IACCTC059552	IACSP955011	SP775158
47	IACCTC059607	IACSP972053	SP963092
48	IACCTC059634	IACSP972053	IACSP953050
49	IACCTC061050	CT943165	IACSP952078
50	IACCTC069708	IACSP962042	SP823697
51	IACCTC069713	IACSP955000	SP87432
52	IACCTC069741	IACSP955000	Unknown
53	IACSP012417	RB835486	RB835486
54	IACSP012430	IAC912195	CT961414
55	IACSP015501	SP913059	SP80185
56	IACSP015519	RB835486	Unknown
57	IACSP018046	R570	Glagah
58	IACSP018082	IAC913093	Glagah
59	IACSP018158	RB835486	Unknown
60	IACSP022067	SP973060	Unknown
61	IACSP022125	SP901161	Unknown
62	IACSP023025	SP924230	Unknown
63	IACSP023168	IAC914216	Unknown
64	IACSP042503	PO8862	Unknown
65	IACSP042504	IACSP955037	Unknown
66	IACSP042509	SP913059	CT961414
67	IACSP042510	IAC913093	CT931231
68	IACSP043123	IACSP953028	RB855453
69	IACSP043148	IACSP953018	IAC911099
70	IACSP043150	IACSP953018	CT014455
71	IACSP043259	IACSP966026	CT961307
72	IACSP045081	IACSP966026	SP775181
73	IACSP046032	IACSP953028	CTC957
74	IACSP046035	SP913059	SP775181
75	IACSP046073	IACSP953028	SP701143
76	IACSP046077	IACSP972109	SP901644
77	IACSP046152	IAC911099	SP88813
78	IACSP933046	SP791011	SP803280
79	IACSP953018	SP842189	CTC9
80	IACSP956114	IAC873187	CTC9638
81	IACSP962008	SP80144	SP832847
82	IACSP963056	SP826108	IACSP933046

83	IACSP963069	SP803280	IACSP933046
84	IACSP963076	SP847017	RB855453
85	IACSP973384	RB855113	IACSP966026
86	IACSP974039	RB835486	SP924221
87	IACSP974048	SP842066	IACSP956114
88	IACSP982053	SP847017	SP801842
89	IACSP983011	IAC913093	CTC9019
90	IACSP993369	SP901616	IACCTC059607
91	IACSP994011	SP842025	RB855453
92	IJ76293	*S. robustum*	IAC863154
93	IN8458	*S. spontaneum*	SP801842
94	IN8488	*S. spontaneum*	Unknown
95	NG57213	Unknown	Unknown
96	RB935744	RB835089	TUC717
97	SP832847	HJ5741	Unknown

Supplementary Table 2. Combinations of primers used for amplified fragment length polymorphism (AFLP) genotyping. Sequences correspond to the three selective nucleotides located at the 3' end of *Eco*RI and *Msp*I primers.

Combination ID	3' Primer sequence	
	***Eco*RI**	***Msp*I**
1	AGA-IRD800	ACA
2	AGA-IRD800	TTG
3	AGA-IRD800	ACC
5	AGA-IRD800	TAG
6	AGA-IRD800	CGC
9	AGA-IRD800	GAA
10	ACA-IRD700	ACA
11	ACA-IRD700	TTG
12	ACA-IRD700	ACC
14	ACA-IRD700	TAG
15	ACA-IRD700	CGC
16	ACA-IRD700	GAG
18	ACA-IRD700	GAA
20	ACC-IRD700	TTG
21	ACC-IRD700	ACC
22	ACC-IRD700	TCG
24	ACC-IRD700	CGC
25	ACC-IRD700	GAG
26	ACC-IRD700	ACT
33	AAC-IRD700	CGC
34	AAC-IRD700	GAG
36	AAC-IRD700	GAA
37	ACG-IRD700	ACA
40	ACG-IRD700	TCG
41	ACG-IRD700	TAG
42	ACG-IRD700	CGC
48	AGG-IRD800	ACC
49	AGG-IRD800	TCG
51	AGG-IRD800	CGC
53	AGG-IRD800	ACT
54	AGG-IRD800	GAA
55	AGG-IRD800	ACA
56	AGG-IRD800	TTG
59	AGG-IRD800	TAG
60	AGG-IRD800	CGC

Supplementary Table 3. Locus name, repetition motif, amplicon size range, annealing temperature (Ta) and sequence of the primers used to amplify the simple sequence repeat (SSR) loci used in the study.

SSR	Motif	Amplicon size range (bp)	Ta (°C)	Forward/Reverse primer sequences
CV29	$(ATCT)_{14}$	85-151	60.0	TCGCGTCCACCAATGTAACC/GCGTGCATCGCTTGTGTCTT
CV37	$(TTTC)_{15}$	114-171	60.0	GGATGGACGACGTGTCCTGG/ATAAAGTGGCCGCTTGGATTGA
CV38	$(CTTTT)_{18}$	93-204	60.0	GAAGCAGGGGCCTCAAGTTG/GTCAAACAGGCGATCTGGCTC
CV60	$(CTCTCC)_{5}$	158-194	60.0	AATCTGCACCCTGCCCTCTC/CAGCTGGAGCATGGATGGAG
CV79	$(CTATAT)_{11}(TATAGA)_{6}$	131-231	60.0	GGCACTGCTGGTGGTTGATTG/TCCCACATCAAGAGGCAGCTA
CV94	$(AAAAAG)_{5}(CGT)_{5}$	182-234	60.0	GGCAGGCCAAGATGAATGAAG/AGCACAGCGGAGGGTACGG
CV106	$(GGC)_{8}$	78-162	60.0	AAACAGAGCATACTCGAGGCC/ACGTTGCTGACGAGGTTTTCC
SCB213	$(TCC)_{6}$	247-337	62.0	AGCCGTCAGGGGTCAGG/ATTCGATGGAGCCTGAGTGAG
SCB312	$(TCC)_{7}$	190-278	64.7	AGTCCGTCGCCGTAATCATCTTG/GCACCTCCTCCTTTCCTTCCTTATT
SCB381	$(TAC)_{8}$	190-257	60.0	TGGAGCTCCGTCTTCTTGTT/GCTAGCCCGTACATTGGGTA
SCB423	$(TTG)_{5}$	250-300	61	CCATGTGGCTTCCTGAAACT/ACAGGCACTTCAAGGGAAGA
SCB436	$(GAG)_{5}$	158-274	60.0	AGTACGCCTGAGTCCTGACG/AGGTGCAAGGGCTGATAGAA

Supplementary Table 4. Distribution of the 97 panel accessions among clusters identified by two hierarchical clustering on principal component (HCPC) analyses performed with best linear unbiased predictor (BLUP) values. Genotypes were divided into three clusters (Q1, Q2 and Q3) based on SCYLV titer determined by RT-qPCR in the first HCPC and into three clusters (SQ1, SQ2 and SQ3) based on SCYL symptom severity and SCYLV titer in the second HCPC.

ID Number	Q Cluster	SQ Cluster	ID Number	Q Cluster	SQ Cluster	ID Number	Q Cluster	SQ Cluster
**1**	Q2	SQ2	**34**	Q2	SQ2	**67**	Q1	SQ1
**2**	Q2	SQ2	**35**	Q1	SQ1	**68**	Q3	SQ2
**3**	Q3	SQ2	**36**	Q2	SQ1	**69**	Q3	SQ2
**4**	Q1	SQ1	**37**	Q1	SQ1	**70**	Q3	SQ3
**5**	Q2	SQ2	**38**	Q1	SQ1	**71**	Q2	SQ2
**6**	Q3	SQ2	**39**	Q1	SQ1	**72**	Q3	SQ2
**7**	Q3	SQ2	**40**	Q1	SQ1	**73**	Q2	SQ2
**8**	Q2	SQ2	**41**	Q1	SQ1	**74**	Q2	SQ2
**9**	Q2	SQ1	**42**	Q3	SQ2	**75**	Q3	SQ2
**10**	Q2	SQ1	**43**	Q1	SQ1	**76**	Q3	SQ2
**11**	Q2	SQ2	**44**	Q1	SQ1	**77**	Q2	SQ2
**12**	Q3	SQ2	**45**	Q2	SQ3	**78**	Q3	SQ2
**13**	Q3	SQ2	**46**	Q3	SQ2	**79**	Q3	SQ2
**14**	Q3	SQ2	**47**	Q3	SQ2	**80**	Q3	SQ2
**15**	Q2	SQ3	**48**	Q2	SQ2	**81**	Q3	SQ2
**16**	Q3	SQ2	**49**	Q2	SQ2	**82**	Q2	SQ2
**17**	Q3	SQ2	**50**	Q3	SQ2	**83**	Q3	SQ3
**18**	Q3	SQ3	**51**	Q3	SQ2	**84**	Q3	SQ2
**19**	Q3	SQ2	**52**	Q3	SQ2	**85**	Q3	SQ2
**20**	Q3	SQ2	**53**	Q3	SQ2	**86**	Q3	SQ2
**21**	Q3	SQ2	**54**	Q2	SQ2	**87**	Q3	SQ2
**22**	Q3	SQ2	**55**	Q3	SQ2	**88**	Q2	SQ2
**23**	Q1	SQ1	**56**	Q2	SQ2	**89**	Q2	SQ2
**24**	Q3	SQ2	**57**	Q3	SQ3	**90**	Q3	SQ2
**25**	Q3	SQ2	**58**	Q2	SQ3	**91**	Q2	SQ2
**26**	Q2	SQ2	**59**	Q3	SQ2	**92**	Q2	SQ1
**27**	Q2	SQ2	**60**	Q2	SQ2	**93**	Q2	SQ2
**28**	Q3	SQ2	**61**	Q3	SQ2	**94**	Q1	SQ1
**29**	Q2	SQ2	**62**	Q2	SQ2	**95**	Q3	SQ2
**30**	Q2	SQ2	**63**	Q2	SQ2	**96**	Q3	SQ2
**31**	Q3	SQ2	**64**	Q2	SQ2	**97**	Q2	SQ2
**32**	Q2	SQ2	**65**	Q2	SQ2			
**33**	Q2	SQ2	**66**	Q2	SQ2			

Supplementary Table 5. P-values associated with Dunn tests comparing the best linear unbiased predictor (BLUP) values of traits between the clusters identified by the two hierarchical clustering on principal components (HCPC) analyses. Asterisks (*) indicate statistical significance ($p < 0.05$).

	Symptom severity	Relative quantification	Absolute quantification
**Q1 vs. Q2**	-	0.0037*	0.0008*
**Q1 vs. Q3**	-	0.0000*	0.0000*
**Q2 vs. Q3**	-	0.0000*	0.0000*
**SQ1 vs. SQ2**	0.0003*	0.0000*	0.0000*
**SQ1 vs. SQ3**	0.0000*	0.0004*	0.0056*
**SQ2 vs. SQ3**	0.0001*	1	0.4037

Supplementary Table 6. Percentage of tag mapping against the nine chosen references using BWA and Bowtie2.

Reference		BWA				Bowtie2			
		Unmapped	Mapped			Unmapped	Mapped		
			1 Time	> 1 Time	Total		1 Time	> 1 Time	Total
***S. bicolor* genome**		71.64%	24.00%	4.36%	28.36%	64.72%	25.99%	9.30%	35.28%
**Methyl-filtered genome**		22.40%	62.50%	15.10%	77.60%	18.13%	49.20%	32.67%	81.87%
**RNA-Seq**		75.34%	24.04%	0.62%	24.66%	72.34%	24.87%	2.79%	27.66%
**De novo assembly**		0.58%	20.78%	78.62%	99.40%	0.13%	4.10%	95.77%	99.87%
**Draft genome**		25.56%	29.52%	44.93%	74.44%	21.91%	22.28%	55.81%	78.09%
**Iso-Seq**		79.61%	7.93%	12.46%	20.39%	62.69%	11.02%	26.29%	37.31%
**Mosaic monoploid genome**		45.34%	39.12%	15.53%	54.66%	41.38%	35.40%	23.22%	58.62%
***S. spontaneum* genome**	**Complete**	11.09%	11.73%	77.18%	88.91%	8.09%	5.46%	86.44%	91.91%
	**Monoploid set**	28.13%	38.34%	33.53%	71.87%	24.21%	32.50%	43.29%	75.79%

Supplementary Table 7. Number of single-nucleotide polymorphisms (SNPs) and insertion and deletion (indel) markers isolated by mapping sequencing tags to each genomic reference.

Reference		Prefiltering			Postfiltering		
		Total markers	SNPs	Indels	Total markers	SNPs	Indels
***S. bicolor* genome**		451,845	288,387	163,458	49,053	28,674	20,379
**Methyl-filtered genome**		1,336,253	870,075	466,178	103,096	60,171	42,925
**RNA-Seq**		417,588	292,943	124,645	43,739	28,934	14,805
**De novo assembly**		120,419	120,419	21,274	133	123	10
**Draft Genome**		681,836	443,727	238,109	41,169	23,568	17,601
**Iso-Seq**		151,783	105,796	45,987	13,147	8,479	4,668
**Mosaic monoploid genome**		882,838	564,585	318,253	74,473	42,750	31,723
***S. spontaneum* genome**	**Complete**	294,525	192,548	101,977	6,184	3,625	2,559
	**Monoploid set**	940,255	574,962	365,293	70,978	38,755	32,223

Supplementary Table 8. Allocation of panel genotypes to clusters identified by population structure analyses. Two separate sets of markers (dominant and codominant) and three clustering methods were employed: a discriminant analysis of principal components (DAPC), a principal component analysis (PCA) followed by k-means and a Bayesian clustering analysis.

ID Number	Dominant DAPC	Dominant k-means	Dominant Bayesian	Codominant DAPC	Codominant k-means	Codominant Bayesian
1	A3	B2	C1	D2	E1	F1
2	A3	B2	C1	D2	E1	F1
3	A3	B2	C1	D3	E2	F1
4	A3	B2	C1	D2	E1	F1
5	A3	B2	C1	D2	E1	F1
6	A3	B1	C1	D2	E1	F1
7	A3	B1	C1	D2	E1	F1
8	A3	B2	C1	D2	E1	F1
9	A1	B3	C2	D2	E1	F1
10	A1	B3	C2	D2	E1	F1
11	A3	B2	C1	D2	E1	F1
12	A3	B2	C1	D2	E1	F1
13	A3	B2	C1	D2	E1	F1
14	A3	B1	C1	D2	E1	F1
15	A3	B2	C1	D2	E1	F1
16	A3	B2	C1	D2	E1	F1
17	A3	B2	C1	D1	E2	F2
18	A3	B2	C1	D2	E1	F1
19	A3	B2	C1	D2	E1	F1
20	A3	B2	C1	D2	E1	F1
21	A3	B2	C1	D2	E1	F1
22	A3	B2	C1	D2	E1	F1
23	A3	B2	C1	D2	E1	F1
24	A3	B2	C1	D2	E1	F1
25	A3	B2	C1	D1	E2	F2
26	A3	B2	C1	D2	E1	F1
27	A3	B2	C1	D2	E1	F1
28	A3	B1	C1	D3	E2	F1
29	A3	B2	C1	D1	E2	F2
30	A3	B1	C1	-	-	-
31	A3	B2	C1	D2	E1	F1
32	A3	B2	C1	D2	E1	F1
33	A3	B2	C1	D2	E1	F1
34	A3	B2	C1	D2	E1	F1
35	A3	B2	C1	D1	E2	F2

36	A3	B2	C1	D2	E1	F1
37	A3	B2	C1	D2	E1	F1
38	A1	B3	C2	D2	E1	F1
39	A1	B3	C2	D2	E1	F1
40	A1	B4	C2	D3	E2	F1
41	A1	B3	C2	D1	E2	F2
42	A1	B4	C2	D2	E1	F1
43	A1	B3	C2	D2	E1	F1
44	A3	B2	C1	D2	E1	F1
45	A3	B2	C1	D2	E1	F1
46	A3	B2	C1	D2	E1	F1
47	A3	B2	C1	D1	E2	F2
48	A3	B2	C1	D2	E1	F1
49	A3	B2	C1	D2	E1	F1
50	A3	B2	C1	D2	E1	F1
51	A3	B2	C1	D2	E1	F1
52	A3	B1	C1	D3	E2	F1
53	A3	B2	C1	D2	E1	F1
54	A3	B2	C1	D2	E1	F1
55	A3	B2	C1	D2	E1	F1
56	A3	B2	C1	D2	E1	F1
57	A3	B2	C1	D2	E1	F1
58	A3	B1	C1	D2	E1	F1
59	A3	B2	C1	D1	E2	F2
60	A3	B1	C1	D2	E1	F1
61	A3	B2	C1	D2	E1	F1
62	A3	B2	C1	D2	E1	F1
63	A3	B2	C1	D2	E1	F1
64	A2	B3	C2	D3	E2	F1
65	A2	B2	C1	D2	E1	F1
66	A2	B3	C2	D2	E1	F1
67	A2	B2	C1	D2	E1	F1
68	A2	B2	C1	D2	E1	F1
69	A2	B3	C2	D2	E1	F1
70	A2	B3	C2	D2	E1	F1
71	A2	B3	C2	D2	E1	F1
72	A2	B2	C1	D2	E1	F1
73	A2	B3	C2	D2	E1	F1
74	-	-	-	D2	E1	F1
75	A2	B3	C2	D2	E1	F1
76	A2	B3	C2	-	-	-
77	A2	B3	C2	D2	E1	F1
78	A2	B3	C2	D2	E1	F1

79	A2	B3	C2	D2	E1	F1
80	A2	B2	C1	D2	E1	F1
81	A2	B3	C2	D2	E1	F1
82	A2	B2	C1	D2	E1	F1
83	A2	B2	C1	D2	E1	F1
84	A2	B3	C2	D2	E1	F1
85	A2	B3	C2	D2	E1	F1
86	A2	B3	C2	D2	E1	F1
87	A2	B2	C1	-	-	-
88	A2	B3	C2	-	-	-
89	A2	B3	C2	D2	E1	F1
90	A2	B3	C2	D1	E2	F2
91	A2	B3	C2	D2	E1	F1
92	A1	B3	C2	D2	E1	F1
93	-	-	-	D2	E1	F1
94	A1	B3	C2	D2	E1	F1
95	A2	B3	C2	-	-	-
96	-	-	-	D2	E1	F1
97	-	-	-	D2	E1	F1

Supplementary Table 9. Dominant markers significantly associated with the three analyzed traits using the fixed and random model circulating probability unification (FarmCPU) method. For each marker-trait association, the trait, $-\log_{10}(p)$ value and the phenotypic variance explained by each marker (adjusted r^2) are provided.

Marker	Trait	$-\log_{10}(p)$	Effect	r^2
MSP_ACT-ECO_AGG-800-0106	Symptom severity	2.79	-1.57	0.116
MSP_TCG-ECO_ACG_700-0240	Relative quantification	2.55	-0.14	0.234
MSP_TCG-ECO_ACG_700-0268	Relative quantification	2.86	0.15	0.249
MSP_TTG-ECO_ACA-700-0133	Relative quantification	2.85	0.19	0.244
MSP_TTG-ECO_ACA-700-0238	Relative quantification	8.42	0.27	0.279
MSP_TTG-ECO_AGA-800-0100	Relative quantification	2.61	0.13	0.213
MSP_ACT-ECO_AGG-800-0116	Absolute quantification	9.09	0.44	0.302
MSP_CGC-ECO_AGG-800-0148	Absolute quantification	4.23	0.21	0.251
MSP_GAA-ECO_AGG-800-0207	Absolute quantification	2.4	0.15	0.099
MSP_TTG-ECO_AAG_800_2-0133	Absolute quantification	2.7	0.25	0.280
MSP_TTG-ECO_ACA-700-0133	Absolute quantification	3.8	0.32	0.222

Supplementary Table 10. Codominant markers significantly associated with the three analyzed traits. For each marker-trait association, the chromosome location, position number, trait, marker-effect model used, $-\log_{10}(p)$ value, effect and polymorphism (reference/alternative alleles) are provided. Effects are not provided for the general and diploidized general models.

Marker	Chromosome	Position	Trait	Model	$-\log_{10}(p)$	Effect	Polymorphism
S1_3322004	1	3322004	Symptom severity	General	6.97	NA	C/T
S1_7029466	1	7029466	Symptom severity	Diplo-general	6.71	NA	T/C
S1_7029466	1	7029466	Symptom severity	Diplo-additive	6.71	-8.18	T/C
S1_7029466	1	7029466	Symptom severity	1-dom-alt	6.71	-8.18	T/C
S1_7029472	1	7029472	Symptom severity	Diplo-general	6.71	NA	-/C
S1_7029472	1	7029472	Symptom severity	Diplo-additive	6.71	-8.18	-/C
S1_7029472	1	7029472	Symptom severity	1-dom-alt	6.71	-8.18	-/C
S1_7029473	1	7029473	Symptom severity	Diplo-general	6.71	NA	-/C
S1_7029473	1	7029473	Symptom severity	Diplo-additive	6.71	-8.18	-/C
S1_7029473	1	7029473	Symptom severity	1-dom-alt	6.71	-8.18	-/C
S1_7029474	1	7029474	Symptom severity	Diplo-general	6.71	NA	-/C
S1_7029474	1	7029474	Symptom severity	Diplo-additive	6.71	-8.18	-/C
S1_7029474	1	7029474	Symptom severity	1-dom-alt	6.71	-8.18	-/C
S1_7029475	1	7029475	Symptom severity	Diplo-general	6.71	NA	-/C
S1_7029475	1	7029475	Symptom severity	Diplo-additive	6.71	-8.18	-/C
S1_7029475	1	7029475	Symptom severity	1-dom-alt	6.71	-8.18	-/C
S1_7029479	1	7029479	Symptom severity	Diplo-general	6.71	NA	C/-
S1_7029479	1	7029479	Symptom severity	Diplo-additive	6.71	-8.18	C/-
S1_7029479	1	7029479	Symptom severity	1-dom-alt	6.71	-8.18	C/-
S1_7029480	1	7029480	Symptom severity	Diplo-general	6.71	NA	C/-
S1_7029480	1	7029480	Symptom severity	Diplo-additive	6.71	-8.18	C/-
S1_7029480	1	7029480	Symptom severity	1-dom-alt	6.71	-8.18	C/-
S1_7029481	1	7029481	Symptom severity	Diplo-general	6.71	NA	C/-
S1_7029481	1	7029481	Symptom severity	Diplo-additive	6.71	-8.18	C/-
S1_7029481	1	7029481	Symptom severity	1-dom-alt	6.71	-8.18	C/-

S1_7029482	1	7029482	Symptom severity	Diplo-general	6.71	NA	A/-
S1_7029482	1	7029482	Symptom severity	Diplo-additive	6.71	-8.18	A/-
S1_7029482	1	7029482	Symptom severity	1-dom-alt	6.71	-8.18	A/-
S1_7029483	1	7029483	Symptom severity	Diplo-general	6.71	NA	T/-
S1_7029483	1	7029483	Symptom severity	Diplo-additive	6.71	-8.18	T/-
S1_7029483	1	7029483	Symptom severity	1-dom-alt	6.71	-8.18	T/-
S1_19840994	1	19840994	Symptom severity	General	6.11	NA	A/G
S1_77494328	1	77494328	Symptom severity	General	6.42	NA	A/-
S1_77494331	1	77494331	Symptom severity	General	6.42	NA	G/C
S2_80240870	2	80240870	Symptom severity	General	6.75	NA	C/T
S2_80240899	2	80240899	Symptom severity	General	7.06	NA	C/T
S2_80240911	2	80240911	Symptom severity	General	6.73	NA	G/C
S2_80240919	2	80240919	Symptom severity	General	6.76	NA	T/-
S2_80240920	2	80240920	Symptom severity	General	6.93	NA	A/T
S3_12947458	3	12947458	Symptom severity	General	8.74	NA	T/C
S3_48163530	3	48163530	Symptom severity	General	7.06	NA	T/C
S3_54754895	3	54754895	Symptom severity	General	6.14	NA	A/G
S4_11629587	4	11629587	Symptom severity	General	6.42	NA	T/C
S4_58593938	4	58593938	Symptom severity	General	7	NA	G/A
S4_62622513	4	62622513	Symptom severity	Diplo-general	6.11	NA	T/-
S4_62622513	4	62622513	Symptom severity	Diplo-additive	6.11	-5.21	T/-
S4_62622513	4	62622513	Symptom severity	1-dom-alt	6.11	-5.21	T/-
S5_78687022	5	78687022	Symptom severity	General	7.53	NA	C/G
S6_19432542	6	19432542	Symptom severity	General	7.41	NA	A/G
S6_20347039	6	20347039	Symptom severity	General	6.53	NA	-/A
S6_58198630	6	58198630	Symptom severity	General	7.09	NA	A/T
S8_10096401	8	10096401	Symptom severity	General	6.88	NA	C/-
S8_10096402	8	10096402	Symptom severity	General	6.88	NA	T/-
S8_10096403	8	10096403	Symptom severity	General	6.88	NA	T/-
S8_10096406	8	10096406	Symptom severity	General	6.88	NA	T/A
S8_10096428	8	10096428	Symptom severity	General	6.23	NA	T/G

SS_190207740	-	190207740	Symptom severity	General	6.88	NA	A/G
S1_11503096	1	11503096	Relative quantification	Diplo-general	4.05	NA	-/T
S1_11503096	1	11503096	Relative quantification	Diplo-additive	4.82	-0.96	-/T
S1_11503097	1	11503097	Relative quantification	Diplo-general	4.05	NA	-/T
S1_11503097	1	11503097	Relative quantification	Diplo-additive	4.82	-0.96	-/T
S1_11503112	1	11503112	Relative quantification	Diplo-general	4.05	NA	T/-
S1_11503112	1	11503112	Relative quantification	Diplo-additive	4.82	-0.96	T/-
S1_11503113	1	11503113	Relative quantification	Diplo-general	4.05	NA	T/-
S1_11503113	1	11503113	Relative quantification	Diplo-additive	4.82	-0.96	A/-
S1_105811826	1	105811826	Relative quantification	Diplo-general	4.17	NA	A/G
S1_105811826	1	105811826	Relative quantification	Diplo-additive	4.17	0.68	A/G
S1_105811826	1	105811826	Relative quantification	1-dom-alt	4.17	0.68	A/G
S2_100207393	2	100207393	Relative quantification	General	4.16	NA	G/C
S2_101252947	2	101252947	Relative quantification	General	4.67	NA	-/A
S2_111395374	2	111395374	Relative quantification	Additive	4.61	-0.17	C/T
S3_5029059	3	5029059	Relative quantification	General	4.07	NA	A/-
S3_5029059	3	5029059	Relative quantification	Additive	4.17	-0.19	A/-
S3_38170436	3	38170436	Relative quantification	General	4.38	NA	C/A
S3_51566876	3	51566876	Relative quantification	General	4.34	NA	G/-
S3_51566877	3	51566877	Relative quantification	General	4.34	NA	C/-
S3_51566897	3	51566897	Relative quantification	General	4.44	NA	T/C
S3_51566905	3	51566905	Relative quantification	General	4.44	NA	T/C
S3_51566929	3	51566929	Relative quantification	General	5	NA	A/C
S8_2650197	8	2650197	Relative quantification	General	4	NA	-/A
SS_100753956	-	100753956	Relative quantification	Additive	4.49	-0.1	C/T
S2_3440535	2	3440535	Absolute quantification	General	4.88	NA	A/T
S2_69603253	2	69603253	Absolute quantification	General	4.25	NA	A/T
S2_69603262	2	69603262	Absolute quantification	General	4.01	NA	A/C
S2_69603265	2	69603265	Absolute quantification	General	4.31	NA	T/A
S2_69603295	2	69603295	Absolute quantification	General	4.31	NA	A/T
S2_121155445	2	121155445	Absolute quantification	General	4.2	NA	A/-

S3_5029059	3	5029059	Absolute quantification	General	4.19	NA	A/-
S7_25132085	7	25132085	Absolute quantification	Diplo-general	4.37	NA	C/T
S7_25132085	7	25132085	Absolute quantification	Diplo-additive	4.37	-1.4	C/T
S7_25132085	7	25132085	Absolute quantification	1-dom-alt	4.37	-1.4	C/T
S7_76514321	7	76514321	Absolute quantification	General	4.32	NA	C/-

Supplementary Table 11. Cross-validation results of the predictive accuracy of each machine learning (ML) model and feature selection (FS) combination employed to predict clustering by SCYLV titer determined by RT-qPCR (Q). The machine learning models tested were adaptive boosting (AB), decision tree (DT), Gaussian naive Bayes (GNB), Gaussian process (GP), K-nearest neighbor (KNN), multilayer perceptron (MLP), random forest (RF) and support vector machine (SVM). FS methods tested were gradient tree boosting (FS1), L1-based FS through a linear support vector classification system (FS2), extremely randomized trees (FS3), univariate FS using ANOVA (FS4) and RF (FS5), in addition to the intersection of markers selected by at least two of all FS methods (Inter1), the intersection of at least two of the three best FS methods (Inter2) and the intersection of the three best FS methods (Inter3).

Model	All markers	FS1	FS2	FS3	FS4	FS5	Inter1	Inter2	Inter3
**AB**	44.35	59.22	53.55	50.33	49.37	49.55	53.94	57.44	63.78
**DT**	39.22	59.02	48.39	48.07	44.83	45.97	50.93	55.72	61.02
**GNB**	44.9	64.82	85.67	58.19	62.29	53.39	76.88	86.17	69.91
**GP**	47.78	51.06	50.64	47.78	47.78	47.78	47.78	51.06	75.79
**KNN**	40.99	67.29	81.28	53.86	65.57	55.45	76.09	86.43	71.67
**MLP**	41.43	69.18	97.64	65.38	84.89	60.05	94.57	95.68	78.72
**RF**	48	76.1	79.58	65	64.63	61.89	74.98	79.52	71.71
**SVM**	49.58	72.35	88.59	63.01	77.72	59.39	84.28	87.82	73.48

Supplementary Table 12. Cross-validation results of the predictive accuracy of each machine learning (ML) model and feature selection (FS) combination employed to predict clustering by SCYLV titer determined by RT-qPCR and SCYL symptom severity (SQ). The machine learning models tested were adaptive boosting (AB), decision tree (DT), Gaussian naive Bayes (GNB), Gaussian process (GP), K-nearest neighbor (KNN), multilayer perceptron (MLP), random forest (RF) and support vector machine (SVM). FS methods tested were gradient tree boosting (FS1), L1-based FS through a linear support vector classification system (FS2), extremely randomized trees (FS3), univariate FS using ANOVA (FS4) and RF (FS5), in addition to the intersection of markers selected by at least two of all FS methods (Inter1), the intersection of at least two of the three best FS methods (Inter2) and the intersection of the three best FS methods (Inter3).

Model	All markers	FS1	FS2	FS3	FS4	FS5	Inter 1	Inter 2	Inter 3
**AB**	69.82	75.05	74.91	72.3	71.96	70.86	72.55	74.24	75.39
**DT**	64.92	80.48	73.1	70.03	69.92	64.72	73.42	75.71	83.08
**GNB**	73.81	78.84	82.2	70.34	79.13	67.71	82.75	87.66	69.32
**GP**	7.91	10.82	11.73	7.91	7.91	7.91	7.91	11.73	78.68
**KNN**	64.45	69.32	75.66	72.95	74.1	72.36	75.28	77.67	78.2
**MLP**	36.32	71.07	96.48	66.51	88.19	60.94	91.19	95.42	84.19
**RF**	73.13	78.12	74.8	73.93	74.5	73.91	76.26	77.42	85.4
**SVM**	73.92	73.92	76.51	73.92	73.01	73.92	75.51	86.49	84.55

Supplementary Table 13. Markers selected by at least two of the three best feature selection methods, considering clustering by SCYLV titer determined by RT-qPCR (Q). For each marker, the polymorphism (reference/alternative alleles) is provided.

Marker	Polymorphism	Marker	Polymorphism
ECO_ACA_700_2-MSP_ACC-0201	0/1	S4_5037630	C/A
ECO_ACG_700_3-MSP_ACA-0350	0/1	S4_51172780	C/T
ECO_ACG_700_3-MSP_CGC-0192	0/1	S4_55959846	G/A
MSP_ACC-ECO_ACC-700-0487	0/1	S4_62067807	G/C
MSP_ACT-ECO_ACC-700-0165	0/1	S4_62094357	A/-
MSP_ACT-ECO_ACC-700-0185	0/1	S4_72511101	G/C
MSP_ACT-ECO_AGG-800-0116	0/1	S5_16631107	A/-
MSP_TAG-ECO_ACG_700_3-0186	0/1	S5_19865296	A/T
MSP_TTG-ECO_ACA-700-0133	0/1	S5_19865299	A/-
MSP_TTG-ECO_ACA-700-0135	0/1	S5_30396509	A/G
MSP_TTG-ECO_ACA-700-0238	0/1	S5_37031999	C/-
SCB381.09	0/1	S5_37032002	C/-
S1_10441059	A/-	S5_37032003	C/-
S1_1129066	G/A	S5_37032005	G/-
S1_13512336	T/C	S5_37032006	C/-
S1_3175304	T/C	S5_37032007	G/-
S1_33374268	T/G	S5_37929370	A/G
S1_33429745	T/C	S5_38045669	C/T
S1_33714180	A/-	S5_39262786	G/C
S1_33714200	A/-	S5_39778394	G/C
S1_36913604	C/T	S5_4018386	G/C
S1_38610862	A/G	S5_40247302	C/G
S1_39200477	A/C	S5_51822173	T/C
S1_3951700	C/T	S5_58262048	C/T
S1_50968980	T/-	S5_58346648	C/T
S1_73987041	C/-	S5_64114690	A/-
S1_75517043	T/-	S5_70115114	A/G
S1_78580392	G/A	S5_77911159	C/G
S1_80885415	-/A	S5_79730136	A/-
S1_84276876	-/T	S5_81637789	T/A
S1_85040557	T/C	S5_83270565	T/C
S1_89312901	T/A	S5_86641489	G/C
S1_89317063	C/-	S5_87002893	G/T
S1_90526407	A/G	S5_87002894	A/G
S1_94787735	T/C	S5_87002896	C/A
S2_101252947	-/A	S5_87002901	A/T
S2_101645661	T/A	S6_23313127	T/C

S2_102692127	-/C	S6_31110903	A/G
S2_10357059	T/-	S6_37262886	G/T
S2_103759601	C/A	S6_39996328	A/-
S2_104765621	A/C	S6_45993856	T/-
S2_107167277	C/T	S6_47989239	G/A
S2_107531177	C/T	S6_49935949	T/C
S2_11407928	T/-	S6_50045923	G/C
S2_11407929	C/-	S6_51715201	A/G
S2_114931317	T/-	S6_54218288	C/T
S2_117480270	A/G	S6_7789087	C/-
S2_11992364	C/T	S6_7789088	C/-
S2_22694123	C/T	S6_88185776	T/-
S2_31743348	T/A	S6_90911017	G/A
S2_3441185	C/G	S6_94271628	A/C
S2_4041569	-/C	S6_97767289	-/C
S2_47286302	C/A	S7_16860739	C/-
S2_580272	T/C	S7_24246331	C/T
S2_67409332	C/-	S7_25494326	T/A
S2_67409335	T/-	S7_28163260	G/C
S2_67409336	T/-	S7_33839422	G/A
S2_67409337	C/-	S7_41636976	A/G
S2_74145864	G/C	S7_49705297	G/T
S2_79667911	A/G	S7_51204011	A/-
S2_81096972	A/T	S7_51204013	C/-
S2_84707411	A/G	S7_52954378	G/A
S2_84898011	G/C	S7_61409653	C/T
S2_86412851	C/G	S7_76845127	C/T
S2_95211978	A/-	S7_8727482	A/C
S2_99027215	T/C	S8_15175965	T/-
S3_12466029	T/A	S8_17563520	T/G
S3_31119563	-/T	S8_21423807	A/C
S3_35454812	-/G	S8_22051935	-/T
S3_36459728	-/G	S8_32042389	A/G
S3_38170436	C/A	S8_35612220	G/-
S3_41060075	T/A	S8_3868588	C/A
S3_43081503	A/T	S8_43975929	G/A
S3_49105147	G/A	S8_49191873	C/T
S3_50218210	T/-	S8_62846201	G/A
S3_5029059	A/-	S8_65429134	A/-
S3_55613831	T/-	SS_108682254	A/T
S3_57815128	C/A	SS_115845288	-/A
S3_59564236	A/-	SS_125997282	G/C
S3_69969353	C/-	SS_162306126	A/G

S3_72076395	C/T	SS_183828723	A/G
S3_73057726	-/C	SS_196174663	C/T
S3_73057731	C/-	SS_213100017	A/G
S3_77376342	T/A	SS_214099396	G/-
S3_77376349	C/G	SS_214099400	-/T
S3_9810328	A/G	SS_229367801	A/G
S4_16437374	T/C	SS_4267291	T/G
S4_16582952	A/G	SS_53385994	G/C
S4_17019688	T/C	SS_53417038	G/A
S4_21976556	C/T	SS_53615857	T/-
S4_22573652	A/G	SS_60091719	G/-
S4_29702262	C/T	SS_60091744	-/C
S4_33241614	-/G	SS_6997624	A/-
S4_3664938	A/T	SS_6997635	-/A
S4_4503306	C/T	SS_6997636	-/A

Supplementary Table 14. Markers selected by at least two of the three best feature selection methods, considering clustering by SCYLV titer determined by RT-qPCR and SCYL symptom severity (SQ). For each marker, the polymorphism (reference/alternative alleles) is provided.

Marker	Polymorphism	Marker	Polymorphism
MSP_ACA-ECO_AAG_800_2-0121	0/1	S3_77376349	C/G
MSP_ACT-ECO_ACC-700-0104	0/1	S4_15496266	A/C
MSP_ACT-ECO_AGG-800-0106	0/1	S4_17037843	T/G
MSP_TTG-ECO_ACA-700-0094	0/1	S4_20005282	C/G
MSP_TTG-ECO_ACA-700-0133	0/1	S4_21970615	G/T
MSP_TTG-ECO_ACA-700-0238	0/1	S4_21970622	A/-
S1_106155638	T/-	S4_26164754	-/G
S1_106155646	G/T	S4_27872858	G/-
S1_106155651	G/T	S4_29702516	G/-
S1_1129066	G/A	S4_38998631	C/T
S1_13277038	A/T	S4_52361599	C/T
S1_15193842	A/G	S4_60441000	G/T
S1_15204569	A/G	S4_67834354	C/T
S1_16467489	-/T	S4_71472623	T/C
S1_21768021	G/A	S4_72511101	G/C
S1_27017708	G/A	S5_12511335	-/C
S1_32004666	G/A	S5_34032519	G/-
S1_33436946	C/-	S5_34032520	A/-
S1_33436948	C/-	S5_41180286	A/-
S1_36984611	A/C	S5_62628785	C/G
S1_55352018	C/T	S5_64114690	A/-
S1_6432921	C/T	S5_70114127	T/-
S1_6432925	-/A	S5_70695514	A/C
S1_6432926	A/-	S5_74260966	G/-
S1_73330330	A/G	S5_78501954	-/A
S1_73898167	A/-	S5_83270565	T/C
S1_75976908	C/T	S5_85563234	A/G
S1_76405126	C/-	S6_16520022	-/T
S1_81690247	C/-	S6_21026466	A/-
S1_96887191	-/C	S6_21026467	G/-
S1_98588359	A/G	S6_21979304	G/A
S2_102692127	-/C	S6_43117749	C/T
S2_10292064	T/A	S6_44425039	C/A
S2_10491272	-/T	S6_51747509	G/T
S2_107167277	C/T	S6_69184546	A/G
S2_10984287	-/G	S7_14264782	A/C
S2_12543228	G/A	S7_21392489	C/T

S2_1695387	G/A	S7_25558360	T/G
S2_20752623	-/G	S7_37524792	T/C
S2_2344737	A/G	S7_40187897	T/C
S2_29082911	T/C	S7_4925034	T/C
S2_32500210	-/T	S7_7265178	T/C
S2_3384263	-/C	S7_78538330	G/A
S2_36266547	-/T	S8_15352646	A/C
S2_62995081	G/A	S8_15352661	C/T
S2_67466872	G/A	S8_17563520	T/G
S2_71898215	C/T	S8_20515788	A/C
S2_78043402	C/A	S8_26548878	C/G
S2_80240920	A/T	S8_39882618	G/T
S2_85812601	T/-	S8_41932797	A/C
S3_19279325	G/C	S8_50905917	C/T
S3_2171611	-/A	S8_59270955	A/G
S3_43081503	A/T	S8_8575896	T/G
S3_49665310	A/G	SS_100090050	A/T
S3_5029059	A/-	SS_185211457	A/G
S3_53391124	G/C	SS_190207740	A/G
S3_53603607	A/C	SS_28567348	G/A
S3_54707120	T/G	SS_53615857	T/-
S3_55712812	T/C	SS_53991168	C/T
S3_74204066	G/-	SS_84033755	A/G

Supplementary Table 15. Annotation of markers associated with the three analyzed traits. Annotation was obtained by aligning 2,000-bp neighboring regions with the genomes of closely related plant species. For each marker, the approach used for association, associated trait, gene, alignment percentage of identity (% ID), alignment E-value and gene ontology annotation are provided.

Marker	Approach	Gene	%ID	E-value	Annotation
SCB381.09	ML_SQ	SbRio.10G317500.1	95.55	0	Peroxidase precursor, putative, expressed
S1_10441059	ML_Q	Sobic.001G494500.2	97.61	5.51E-118	Kinase, pfkB family, putative, expressed
S1_105811826	GWAS_Rel	Sobic.001G053100.2	78.73	4.08E-149	Expressed protein
S1_13277038	ML_SQ	Oropetium_20150105_00333A	97.17	2.20E-42	Prenyltransferase, putative, expressed
S1_13512336	ML_Q	SbRio.01G504900.1	99.07	7.86E-47	OsSCP11 - Putative Serine Carboxypeptidase homologue, expressed
S1_15193842	ML_SQ	Sobic.001G464100.1	98.83	3.48E-80	Cytochrome b5-like Heme/Steroid binding domain containing protein, expressed
S1_15204569	ML_SQ	Zm00001d048138_T026	97.85	7.98E-37	BTBN4 - Bric-a-Brac, Tramtrack, Broad Complex BTB domain with nonphototropic hypocotyl 3 NPH3 domain, expressed
S1_16467489	ML_SQ	Sobic.001G452600.1	98.47	7.02E-127	Leucine-rich repeat family protein, putative, expressed
S1_19840994	GWAS_Sym	Sobic.001G417400.1	94.54	5.66E-98	RNA recognition motif containing protein, putative, expressed
S1_3175304	ML_Q	Sobic.001G532200.1	96.18	4.20E-129	OsRhmbd7 - Putative Rhomboid homologue, expressed
S1_32004666	ML_SQ	Sobic.001G346600.1	98.03	5.91E-68	Kinesin heavy chain isolog, putative, expressed
S1_33374268	ML_Q	Pavir.3KG265854.1	83.33	7.64E-67	Transposon protein, putative, unclassified, expressed
S1_33436946	ML_SQ	SbRio.01G362100.1	91.46	0	Cytochrome P450, putative, expressed
S1_33436948	ML_SQ	SbRio.01G362100.1	91.46	0	Cytochrome P450, putative, expressed
S1_38610862	ML_Q	Sobic.001G305100.1	98.23	6.08E-48	Pectinacetylesterase domain containing protein, expressed
S1_3951700	ML_Q	Sobic.001G528000.1	95.19	6.17E-38	SNARE domain containing protein, putative, expressed
S1_55352018	ML_SQ	SbRio.06G054100.1	99.03	4.73E-44	Vacuolar-sorting receptor precursor, putative, expressed
S1_6432921	ML_SQ	Zm00001d027607_T001	98.31	3.63E-50	Cysteine-rich repeat secretory protein precursor, putative, expressed

S1_6432925	ML_SQ	Zm00001d027607_T001	98.31	3.63E-50	Cysteine-rich repeat secretory protein precursor, putative, expressed
S1_6432926	ML_SQ	Zm00001d027607_T001	98.31	3.63E-50	Cysteine-rich repeat secretory protein precursor, putative, expressed
S1_7029466	GWAS_Sym	SbRio.01G540100.1	94.51	0	GATA zinc finger domain containing protein, expressed
S1_7029472	GWAS_Sym	SbRio.01G540100.1	94.61	0	GATA zinc finger domain containing protein, expressed
S1_7029473	GWAS_Sym	SbRio.01G540100.1	94.61	0	GATA zinc finger domain containing protein, expressed
S1_7029474	GWAS_Sym	SbRio.01G540100.1	94.61	0	GATA zinc finger domain containing protein, expressed
S1_7029475	GWAS_Sym	SbRio.01G540100.1	94.61	0	GATA zinc finger domain containing protein, expressed
S1_7029479	GWAS_Sym	SbRio.01G540100.1	94.58	0	GATA zinc finger domain containing protein, expressed
S1_7029480	GWAS_Sym	SbRio.01G540100.1	94.57	0	GATA zinc finger domain containing protein, expressed
S1_7029481	GWAS_Sym	SbRio.01G540100.1	94.56	0	GATA zinc finger domain containing protein, expressed
S1_7029482	GWAS_Sym	SbRio.01G540100.1	94.55	0	GATA zinc finger domain containing protein, expressed
S1_7029483	GWAS_Sym	SbRio.01G540100.1	94.54	0	GATA zinc finger domain containing protein, expressed
S1_73330330	ML_SQ	Sobic.001G217200.2	98.94	4.44E-89	START domain containing protein, expressed
S1_73898167	ML_SQ	Sobic.001G214000.1	98.48	5.17E-163	Dicer, putative, expressed
S1_75976908	ML_SQ	SbRio.01G476900.1	100.00	2.87E-36	AP2/EREBP transcription factor BABY BOOM, putative, expressed
S1_76405126	ML_SQ	Zm00001d032575_T013	97.70	1.73E-33	Expressed protein
S1_77494328	GWAS_Sym	Sobic.001G200200.2	97.80	1.03E-35	Tetratricopeptide repeat, putative, expressed
S1_77494331	GWAS_Sym	Sobic.001G200200.2	97.80	1.03E-35	Tetratricopeptide repeat, putative, expressed
S1_78580392	ML_Q	Sobic.001G192000.1	96.32	0	Autophagy protein 9, putative, expressed
S1_80885415	ML_Q	Sobic.001G186700.1	94.25	0	Zinc finger C-x8-C-x5-C-x3-H type family protein
S1_81690247	ML_SQ	Sobic.001G185200.1	96.43	4.73E-44	Expressed protein
S1_85040557	ML_Q	Sobic.001G163300.2	96.23	0	Zinc finger protein-related, putative, expressed
S1_90526407	ML_Q	Sobic.001G122900.1	95.68	1.00E-55	Expressed protein
S1_96887191	ML_SQ	Sobic.001G106500.1	88.68	5.95E-63	Expressed protein

S2_100207393	GWAS_Rel	Sobic.008G107100.1	93.23	0	Cytochrome P450, putative, expressed
S2_101645661	ML_Q	SbRio.08G127900.1	95.20	6.08E-48	rhoGAP domain containing protein, expressed
S2_102692127	ML_Q/ML_SQ	Seita.6G074900.1	91.20	7.81E-52	BTBN17 - Bric-a-Brac, Tramtrack, Broad Complex BTB domain with nonphototropic hypocotyl 3 NPH3 and coiled-coil domains, expressed
S2_10357059	ML_Q	GRMZM2G017080_T02	96.15	4.77E-39	Hydrolase protein, putative, expressed
S2_104765621	ML_Q	Sobic.008G132550.1	87.97	0	Pleiotropic drug resistance protein, putative, expressed
S2_10491272	ML_SQ	Sobic.002G380700.1	94.82	1.19E-114	Expressed protein
S2_107167277	ML_Q/ML_SQ	Sobic.008G141100.1	100.00	4.73E-44	Retrotransposon protein, putative, unclassified, expressed
S2_111395374	GWAS_Rel	Sobic.008G156600.1	95.53	9.35E-106	Leucine Rich Repeat family protein, expressed
S2_12543228	ML_SQ	Sobic.002G363500.2	100.00	4.77E-39	RNA methyltransferase, TrmH family protein, putative, expressed
S2_1695387	ML_SQ	Zm00001d007241_T001	97.94	4.77E-39	KIP1, putative, expressed
S2_20752623	ML_SQ	Sobic.002G312800.1	97.75	9.68E-81	WD domain, G-beta repeat domain containing protein, expressed
S2_2344737	ML_SQ	Sobic.002G426800.1	93.87	0	Zinc finger, C3HC4 type domain containing protein, expressed
S2_29082911	ML_SQ	Sobic.002G259000.1	99.02	5.66E-98	Ribosomal protein L7Ae, putative, expressed
S2_31743348	ML_Q	SbRio.02G255400.1	93.50	0	Nucleolar GTP-binding protein 1, putative, expressed
S2_32500210	ML_SQ	Sobic.002G237800.2	100.00	3.68E-40	NOL1/NOP2/sun family protein, putative, expressed
S2_36266547	ML_SQ	Pavir.2NG346303.1	94.18	4.29E-114	Expressed protein
S2_4041569	ML_Q	Sobic.002G415100.2	92.18	5.95E-63	POEI50 - Pollen Ole e I allergen and extensin family protein precursor, expressed
S2_580272	ML_Q	Thint.06G0495000.1	96.77	1.01E-50	MYB family transcription factor, putative, expressed
S2_62995081	ML_SQ	Sobic.002G124900.1	97.12	2.85E-41	ATP synthase D chain, mitochondrial
S2_67409332	ML_Q	Sobic.002G108000.1	94.22	7.38E-92	Myosin, putative, expressed
S2_67409337	ML_Q	Sobic.002G108000.1	94.22	7.38E-92	Myosin, putative, expressed
S2_67466872	ML_SQ	Sobic.002G106700.1	91.85	0	OsWAK75 - OsWAK receptor-like protein kinase, expressed
S2_74145864	ML_Q	Sobic.002G081500.5	92.17	1.67E-58	Glycerol-3-phosphate dehydrogenase, putative, expressed

S2_78043402	ML_SQ	SbRio.02G066000.1	97.22	1.57E-98	OsSub3 - Putative Subtilisin homologue, expressed
S2_79667911	ML_Q	SbRio.02G057300.1	89.06	0	Rf1, mitochondrial precursor, putative, expressed
S2_85812601	ML_SQ	Sobic.002G019750.1	88.94	2.11E-72	OsFBX234 - F-box domain containing protein, expressed
S2_86412851	ML_Q	SbRio.02G018600.1	92.54	3.31E-115	Expressed protein
S3_19279325	ML_SQ	Sobic.003G287000.1	99.09	6.08E-48	BURP domain containing protein, expressed
S3_2171611	ML_SQ	Sobic.003G395400.1	99.30	1.48E-143	BTBZ2 - Bric-a-Brac, Tramtrack, and Broad Complex BTB domain with TAZ zinc finger and Calmodulin-binding domains, expressed
S3_35454812	ML_Q	Zm00001d011212_T001	93.14	1.73E-33	Helix-loop-helix DNA-binding domain containing protein, expressed
S3_41060075	ML_Q	Brasy7G095300.1	89.72	0	Expressed protein
S3_48163530	GWAS_Sym	SbRio.03G158900.1	86.38	0	OsFBX103 - F-box domain containing protein, expressed
S3_49105147	ML_Q	Sobic.003G146900.1	99.20	2.79E-56	Lipase class 3 family protein, putative, expressed
S3_50218210	ML_Q	SbRio.03G148100.1	97.24	0	Receptor-like protein kinase 5 precursor, putative, expressed
S3_54707120	ML_SQ	SbRio.03G120100.1	97.14	0	Lipase, putative, expressed
S3_54754895	GWAS_Sym	SbRio.03G120000.1	93.77	5.13E-168	GCRP6 - Glycine and cysteine rich family protein precursor, putative, expressed
S3_55613831	ML_Q	Zm00001d040210_T001	98.12	3.38E-100	Protein of unknown function (DUF 3339)
S3_57815128	ML_Q	Sobic.003G101500.1	93.75	0	DNAJ domain containing protein, expressed
S3_72076395	ML_Q	Oropetium_20150105_17951A	97.39	2.19E-47	BES1/BZR1 homolog protein, putative, expressed
S3_74204066	ML_SQ	Sobic.003G013100.1	92.57	2.17E-52	GDSL-like lipase/acylhydrolase, putative, expressed
S4_11629587	GWAS_Sym	Sobic.004G242500.1	95.95	9.96E-61	Glycosyl hydrolase family 47 domain contain protein, expressed
S4_15496266	ML_SQ	Sobic.004G267400.2	98.59	4.60E-64	Basic helix-loop-helix, putative, expressed
S4_16437374	ML_Q	Sobic.004G274000.1	95.94	0	STE_MEK_ste7_MAP2K.4 - STE kinases include homologs to sterile 7, sterile 11 and sterile 20 from yeast, expressed
S4_16582952	ML_Q	Zm00001d017699_T001	92.08	1.63E-73	Glycosyl hydrolases family 16, putative, expressed
S4_17037843	ML_SQ	Sobic.004G276900.2	98.14	1.87E-157	Leucyl-tRNA synthetase, cytoplasmic, putative, expressed
S4_20005282	ML_SQ	Sobic.004G296500.2	95.58	6.12E-43	Serine esterase family protein, putative, expressed
S4_21970615	ML_SQ	Sobic.004G224350.1	90.41	2.62E-101	Stage II sporulation protein E, putative, expressed

S4_21970622	ML_SQ	Sobic.004G224350.1	90.64	3.36E-105	Stage II sporulation protein E, putative, expressed
S4_26164754	ML_SQ	Pahal.1G292400.1	97.30	3.66E-45	Expressed protein
S4_29702262	ML_Q	SbRio.04G200400.1	98.14	1.17E-129	Transporter family protein, putative, expressed
S4_29702516	ML_SQ	SbRio.04G200400.1	98.14	1.17E-129	Transporter family protein, putative, expressed
S4_33241614	ML_Q	Sobic.004G165600.1	92.04	1.33E-34	Glycosyl hydrolases family 17, putative, expressed
S4_3664938	ML_Q	SbRio.04G366500.1	96.36	9.28E-111	Uncharacterized protein yqjG, putative, expressed
S4_38998631	ML_SQ	Sobic.004G148100.1	95.76	1.02E-45	Cupin domain containing protein, expressed
S4_4503306	ML_Q	Zm00001d018419_T001	95.76	1.02E-45	Expressed protein
S4_5037630	ML_Q	Sobic.004G333100.1	98.85	7.48E-82	Expressed protein
S4_52361599	ML_SQ	Sobic.003G167000.1	89.81	4.70E-49	Protein phosphotase protein, putative, expressed
S4_60441000	ML_SQ	Sobic.004G070800.1	98.86	4.20E-129	TIC21, putative, expressed
S4_62067807	ML_Q	Zm00001d053733_T001	80.98	2.07E-87	Glycosyl hydrolases family 17, putative, expressed
S4_62622513	GWAS_Sym	HORVU6Hr1G031450.3	97.09	1.02E-40	OsSPL4 - SBP-box gene family member, expressed
S4_67834354	ML_SQ	SbRio.01G107700.1	94.32	0	gp176, putative, expressed
S4_71472623	ML_SQ	Sobic.004G014100.1	98.35	1.24E-84	Sialyltransferase family domain containing protein, expressed
S4_72511101	ML_Q/ML_SQ	HORVU2Hr1G108070.13	97.73	4.80E-34	Glycosyltransferase protein, putative, expressed
S5_30396509	ML_Q	Zm00001d025474_T003	98.37	5.51E-118	Chloride channel C
S5_34032519	ML_SQ	Sobic.006G074900.1	95.03	0	Transposon protein, putative, Mutator subclass, expressed
S5_34032520	ML_SQ	Sobic.006G074900.1	95.02	0	Transposon protein, putative, Mutator subclass, expressed
S5_38045669	ML_Q	Sobic.006G058800.1	99.49	0	tRNA synthetase class II core domain containing protein, expressed
S5_51822173	ML_Q	SbRio.06G023600.1	98.36	1.98E-117	ABC transporter family protein, putative, expressed
S5_58262048	ML_Q	SbRio.06G005000.1	94.00	2.02E-102	Erythronate-4-phosphate dehydrogenase domain containing protein, expressed
S5_70695514	ML_SQ	Zm00001d049007_T001	97.86	3.34E-110	PHD finger protein, putative, expressed
S5_83270565	ML_Q/ML_SQ	Sobic.005G039500.1	100.00	2.19E-47	Signal recognition particle 54 kDa protein, putative, expressed
S5_85563234	ML_SQ	Sobic.005G029100.2	98.79	7.54E-77	PP2A regulatory subunit TAP46, putative, expressed
S5_87002893	ML_Q	Sobic.005G020700.1	96.70	4.80E-34	MATE efflux family protein, putative, expressed

S5_87002894	ML_Q	Sobic.005G020700.1	96.70	4.80E-34	MATE efflux family protein, putative, expressed
S5_87002896	ML_Q	Sobic.005G020700.1	96.70	4.80E-34	MATE efflux family protein, putative, expressed
S5_87002901	ML_Q	Sobic.005G020700.1	96.70	4.80E-34	MATE efflux family protein, putative, expressed
S6_16520022	ML_SQ	Sobic.007G152000.1	98.77	8.03E-32	Activator of 90 kDa heat shock protein ATPase homolog, putative, expressed
S6_19432542	GWAS_Sym	Pahal.6G214300.1	81.90	7.92E-42	STE_MEK_ste7_MAP2K.8 - STE kinases include homologs to sterile 7, sterile 11 and sterile 20 from yeast, expressed
S6_20347039	GWAS_Sym	Pahal.6G208600.1	96.65	5.58E-108	Copper transport protein family
S6_39996328	ML_Q	Sobic.009G009500.1	92.89	7.43E-87	Inhibitor I family protein, putative, expressed
S6_43117749	ML_SQ	Sobic.007G092500.1	86.81	7.22E-107	Expressed protein
S6_45993856	ML_Q	Sobic.007G085400.1	90.85	0	NBS-LRR disease resistance protein, putative, expressed
S6_47989239	ML_Q	Sobic.007G079300.1	96.73	0	Proteasome/cyclosome repeat containing protein, expressed
S6_49935949	ML_Q	Sobic.007G073700.1	97.12	0	Nucleoside transporter, putative, expressed
S6_50045923	ML_Q	Sobic.007G073400.1	97.37	9.61E-86	SHR5-receptor-like kinase, putative, expressed
S6_51715201	ML_Q	Sobic.007G069700.1	98.96	3.68E-40	Nucleolin, putative, expressed
S6_69184546	ML_SQ	Sobic.007G007000.1	98.13	7.59E-72	DUF250 domain containing protein, putative, expressed
S6_7789087	ML_Q	Sobic.007G198400.1	87.65	6.97E-132	BTB/POZ domain containing protein, expressed
S6_7789088	ML_Q	Sobic.007G198400.1	87.65	6.97E-132	BTB/POZ domain containing protein, expressed
S6_94271628	ML_Q	Sobic.005G194000.2	96.21	6.04E-53	DUF1399 containing protein, putative, expressed
S6_97767289	ML_Q	Sobic.005G203500.1	87.07	0	LZ-NBS-LRR class RGA, putative, expressed
S7_14264782	ML_SQ	Sobic.009G174400.7	97.56	7.81E-52	Protein of unknown function (DUF3133)
S7_16860739	ML_Q	Sobic.009G161401.1	96.91	2.22E-37	Expressed protein
S7_24246331	ML_Q	Zm00001d038089_T002	98.11	4.73E-44	Integral membrane transporter family protein, putative, expressed
S7_25132085	GWAS_Abs	Sobic.009G121100.2	91.26	5.87E-73	Plant calmodulin-binding protein-related
S7_25558360	ML_SQ	Zm00001d037864_T030	97.58	1.63E-73	Tetratricopeptide repeat containing protein, putative, expressed
S7_28163260	ML_Q	Sobic.009G108700.2	96.81	1.03E-35	NADP-dependent malic enzyme, chloroplast precursor, putative, expressed
S7_33839422	ML_Q	GRMZM2G045892_T01	97.92	1.71E-38	CBS domain containing protein, expressed

S7_40187897	ML_SQ	Sobic.001G399200.2	89.36	7.92E-42	Kinesin motor domain containing protein, expressed
S7_41636976	ML_Q	Sobic.009G065200.2	96.52	0	Zinc finger family protein, putative, expressed
S7_4925034	ML_SQ	Sevir.3G147201.1	97.93	1.65E-63	WRKY16, expressed
S7_49705297	ML_Q	Sobic.009G035100.1	96.20	2.75E-66	Thioesterase family protein, putative, expressed
S7_51204011	ML_Q	GRMZM5G800780_T01	95.18	0	Cytochrome b6, putative, expressed
S7_51204013	ML_Q	GRMZM5G800780_T01	95.18	0	Cytochrome b6, putative, expressed
S7_61409653	ML_Q	Pahal.3G419900.1	88.98	1.33E-34	UDP-glucoronosyl/UDP-glucosyl transferase, putative, expressed
S7_8727482	ML_Q	Sobic.009G204800.1	97.86	2.77E-61	Senescence-induced receptor-like serine/threonine-protein kinase precursor, putative, expressed
S8_10096401	GWAS_Sym	Sobic.010G230300.1	96.75	3.63E-50	Galactosyltransferase, putative, expressed
S8_10096402	GWAS_Sym	Sobic.010G230300.1	96.77	1.01E-50	Galactosyltransferase, putative, expressed
S8_10096403	GWAS_Sym	Sobic.010G230300.1	96.80	2.81E-51	Galactosyltransferase, putative, expressed
S8_10096406	GWAS_Sym	Sobic.010G230300.1	96.88	6.04E-53	Galactosyltransferase, putative, expressed
S8_10096428	GWAS_Sym	Sobic.010G230300.1	97.33	3.56E-65	Galactosyltransferase, putative, expressed
S8_15352646	ML_SQ	Sobic.010G204400.1	99.23	4.63E-59	Phox domain-containing protein, putative, expressed
S8_15352661	ML_SQ	Sobic.010G204400.1	99.23	4.63E-59	Phox domain-containing protein, putative, expressed
S8_17563520	ML_Q/ML_SQ	Zm00001d046725_T001	92.48	5.62E-103	D-mannose binding lectin family protein, expressed
S8_20515788	ML_SQ	Sobic.010G180400.1	100.00	7.38E-92	Myristoyl-acyl carrier protein thioesterase, chloroplast precursor, putative, expressed
S8_22051935	ML_Q	Sobic.010G172600.1	93.20	0	Expressed protein
S8_26548878	ML_SQ	Sobic.010G160500.4	98.91	6.17E-38	DEAD-box ATP-dependent RNA helicase, putative, expressed
S8_35612220	ML_Q	Zm00001d052404_T002	87.50	3.71E-35	ELMO/CED-12 family protein, putative, expressed
S8_39882618	ML_SQ	Sobic.010G131300.2	97.79	1.17E-129	ABB1 - Ankyrin repeat region with 2 Bric-a-Brac, Tramtrack, Broad Complex BTB domains, expressed
S8_41932797	ML_SQ	Sobic.010G124300.1	96.77	2.04E-97	Oxidoreductase, short chain dehydrogenase/reductase family domain containing protein, expressed
S8_59270955	ML_SQ	SbRio.10G053600.1	98.21	7.54E-77	Tyrosylprotein sulfotransferase
S8_62846201	ML_Q	Sobic.010G031300.1	98.31	1.01E-50	Auxin-induced protein 5NG4, putative, expressed

S8_8575896	ML_SQ	Sobic.010G239600.2	98.45	2.79E-56	RNA polymerase I specific transcription initiation factor RRN3 family protein, putative, expressed
SS_100090050	ML_SQ	Zm00001d045565_T001	95.04	1.02E-45	Phosphoglycolate phosphatase, plasmid, putative, expressed
SS_108682254	ML_Q	Sobic.005G028100.1	99.17	4.67E-54	Nucleolar complex protein 2, putative, expressed
SS_115845288	ML_Q	Sobic.004G276700.1	92.48	1.32E-44	Transposon protein, putative, unclassified, expressed
SS_185211457	ML_SQ	Sobic.002G140800.2	98.75	2.74E-31	RNA pseudouridine synthase, putative, expressed
SS_196174663	ML_Q	Sobic.010G134500.1	97.60	6.04E-53	Exonuclease, putative, expressed
SS_213100017	ML_Q	SbRio.07G035500.1	91.32	0	Pentatricopeptide repeat domain containing protein, putative, expressed
SS_229367801	ML_Q	Sobic.002G296800.3	92.17	2.11E-37	Cell cycle control protein, putative, expressed
SS_28567348	ML_SQ	SbRio.05G003000.1	97.46	3.24E-130	Double Clp-N motif-containing P-loop nucleoside triphosphate hydrolases superfamily protein
SS_4267291	ML_Q	Sobic.004G290000.1	100.00	3.24E-95	OsSub19 - Putative Subtilisin homologue, expressed
SS_53615857	ML_Q/ML_SQ	Sobic.008G082000.1	92.31	1.31E-49	RWP-RK domain-containing protein, putative, expressed
SS_84033755	ML_SQ	Sevir.7G299100.4	84.07	2.61E-66	Transposon protein, putative, Pong subclass, expressed

Supplementary Figures

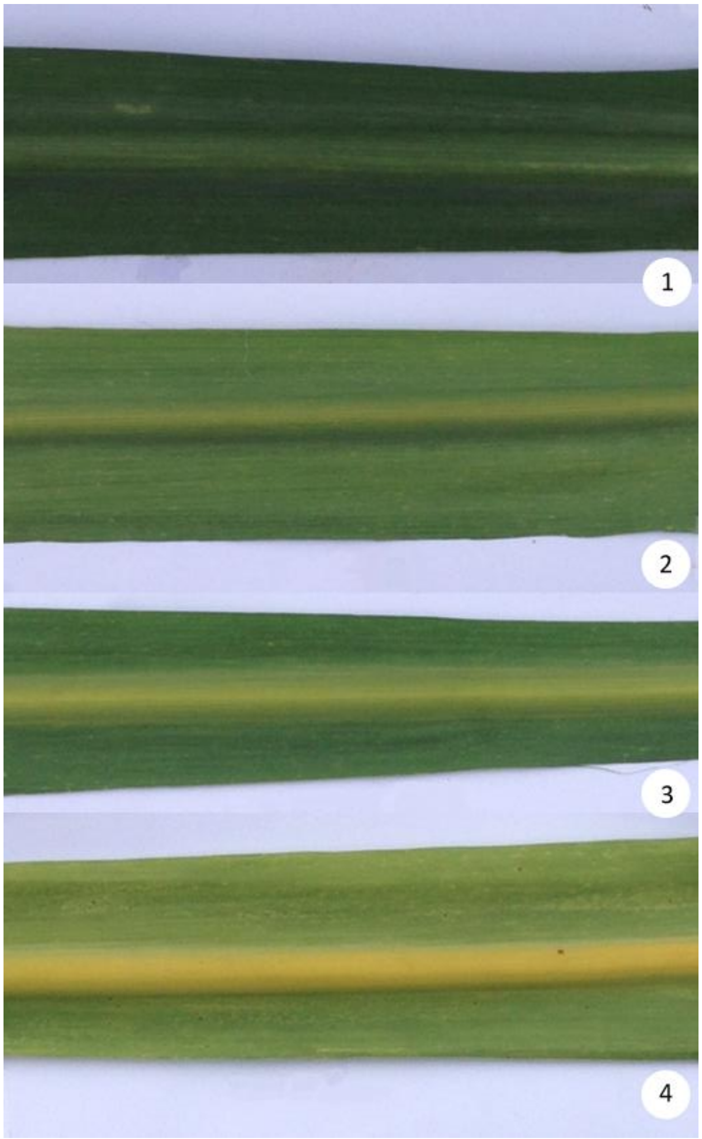

Supplementary Fig. 1 Diagram of the scoring scale for the assessment of sugarcane yellow leaf symptom severity. (1) Green leaf with no symptoms; (2) slight yellowing of the abaxial midrib, with a discrete or no progression to the leaf blade; (3) intense yellowing of the abaxial midrib and a more pronounced yellowing of the leaf blade; (4) intense yellowing of both abaxial midrib and leaf blade. Adapted from Burbano et al. (2021)

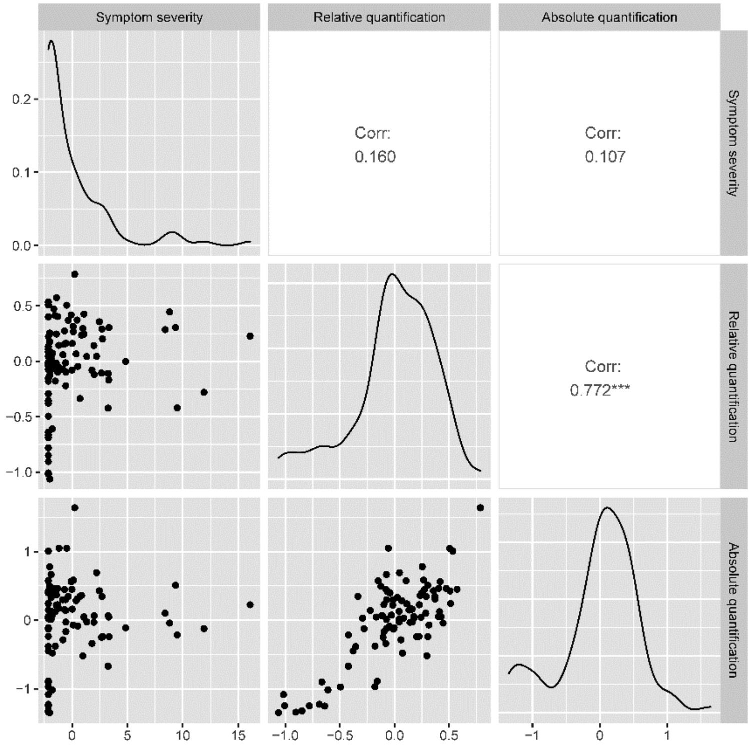

Supplementary Fig. 2 Distributions of BLUP values and correlations between the three traits analyzed.

Asterisks (***) indicate statistical significance ($p < 0.0005$)

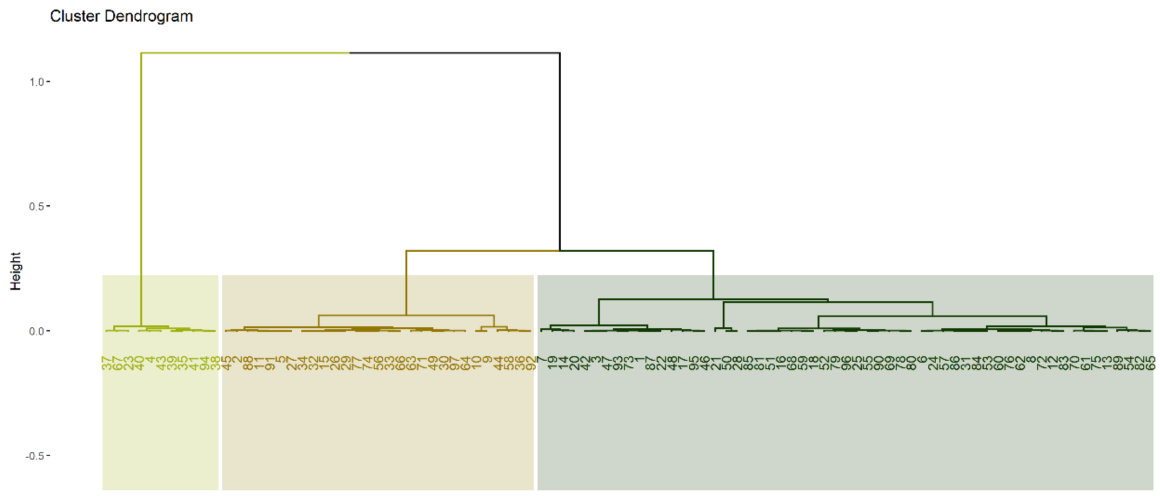

Supplementary Fig. 3 Cluster dendrogram generated in the hierarchical clustering on principal components (HCPC) analysis using the BLUPs of SCYLV titer determined by RT-qPCR. A division into three clusters (Q1/light green, Q2/brown and Q3/dark green) was considered

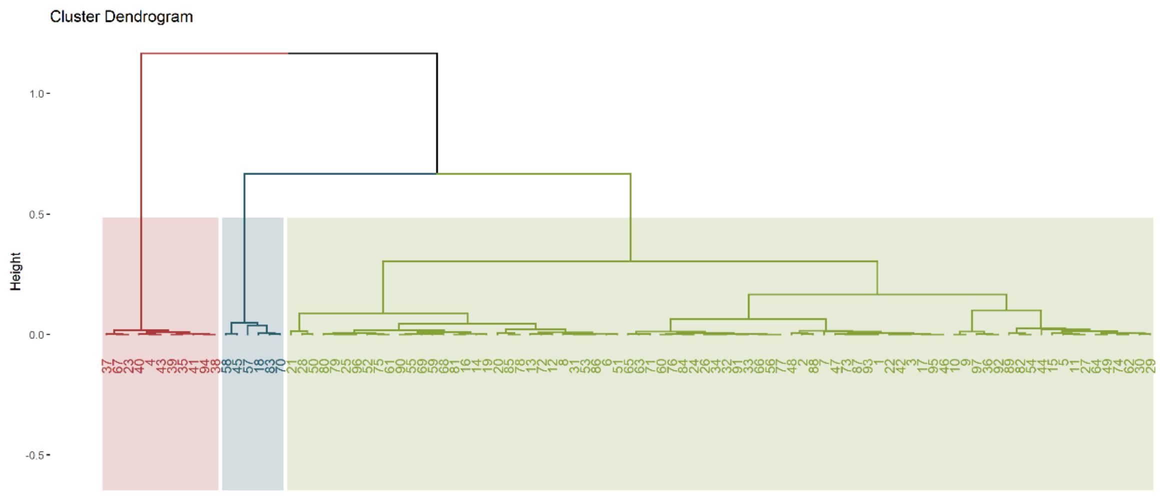

Supplementary Fig. 4 Cluster dendrogram generated in the hierarchical clustering on principal components (HCPC) analysis using BLUPs of SCYL symptom severity and SCYLV titer determined by RT-qPCR. A division into three clusters (SQ1/red, SQ2/green and SQ3/blue) was considered

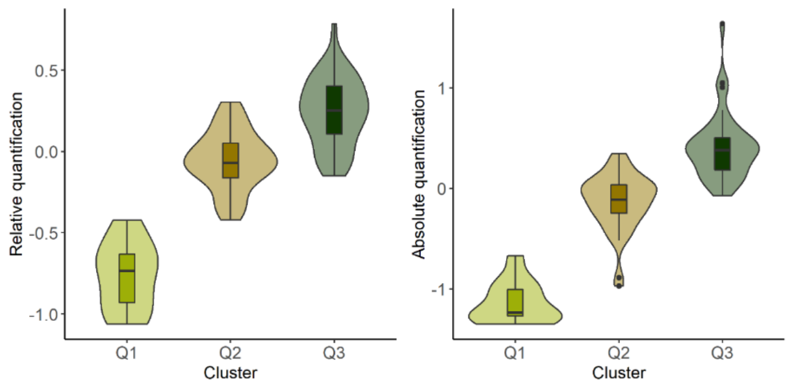

Supplementary Fig. 5 Boxplots depicting the distributions of BLUP values of SCYLV titer determined by relative and absolute quantifications among the three clusters identified in the hierarchical clustering on principal components (HCPC) using the BLUPs of SCYLV titer determined by RT-qPCR. A division into three clusters (Q1, Q2 and Q3) was considered

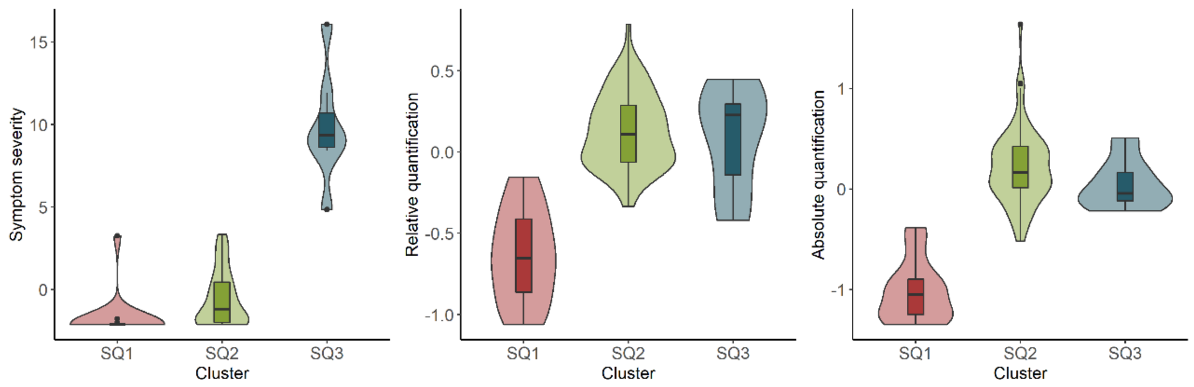

Supplementary Fig. 6 Boxplots depicting the distributions of BLUP values for the three analyzed traits among the three clusters identified in the hierarchical clustering on principal components (HCPC) using BLUPs of SCYL symptom severity and SCYLV titer determined by RT-qPCR. A division into three clusters (SQ1, SQ2 and SQ3) was considered

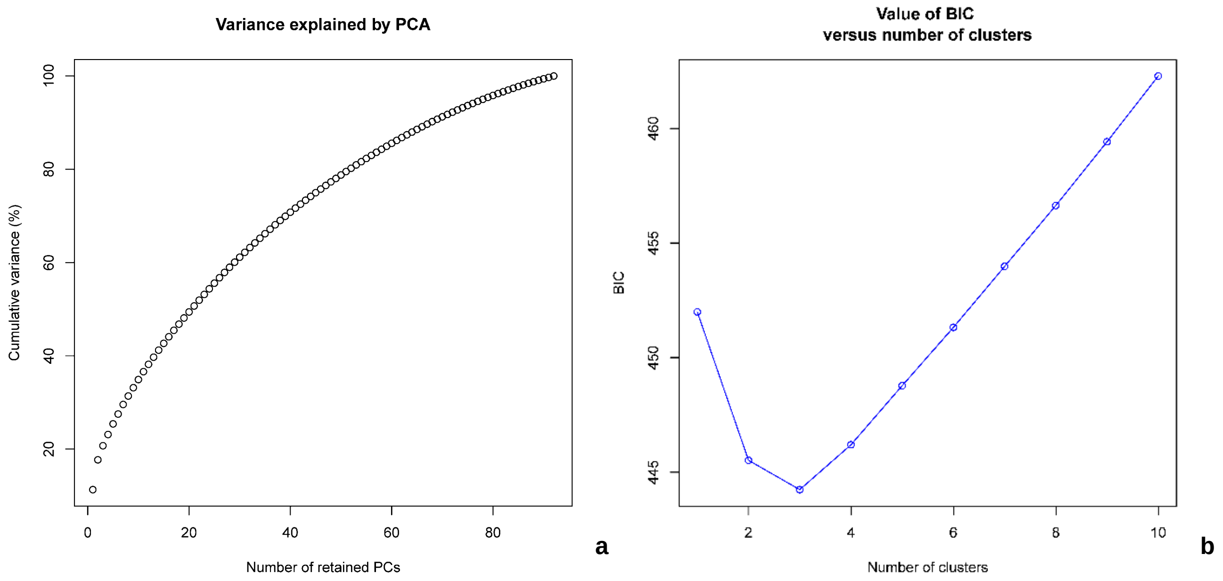

Supplementary Fig. 7 Graphical output of methods employed for the determination of the optimal number of genetic clusters in the panel based on a discriminant analysis of principal components (DAPC) using 622 dominant markers. (a) Cumulative variance explained by the principal components in the analysis. **(b)** Bayesian information criterion (BIC) versus number of clusters in k-means clustering

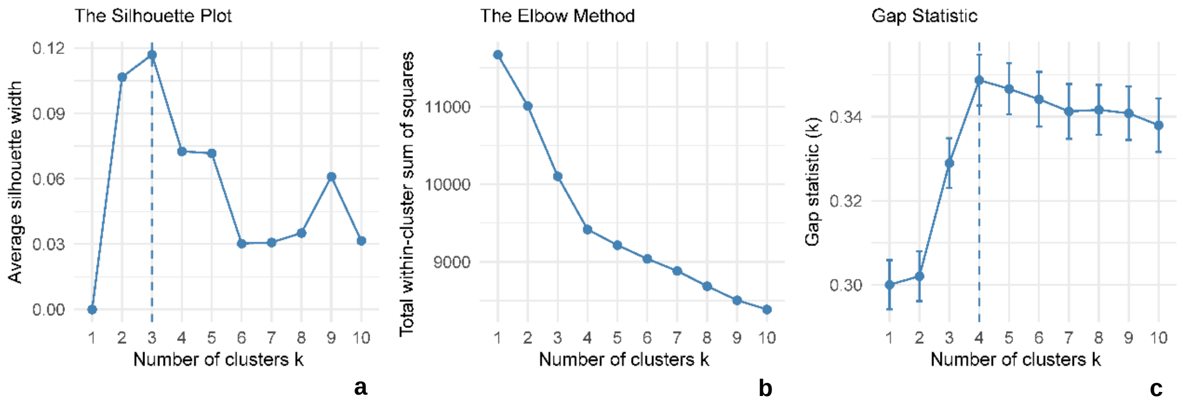

Supplementary Fig. 8 Graphical output of methods used for determining the optimal number of clusters in the panel from a principal component analysis (PCA) using 622 dominant markers. (a) Silhouette plot. **(b)** Elbow method. **(c)** Gap statistic

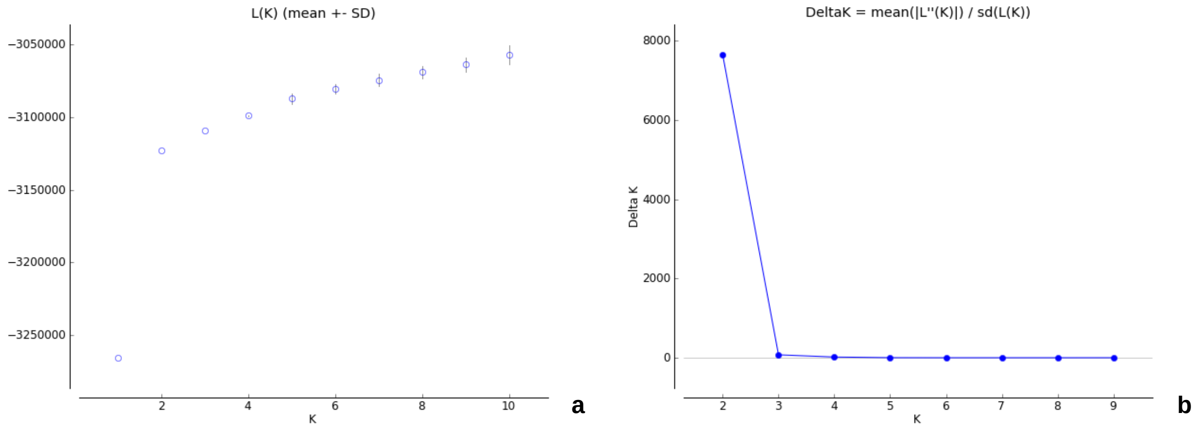

Supplementary Fig. 9 Graphical output of methods used for the determination of the optimal number of genetic clusters in the panel based on the STRUCTURE Bayesian clustering analysis using 622 dominant markers. (a) Mean LnP(D) for K = 1-10. **(b)** Mean ΔK for K = 1-10

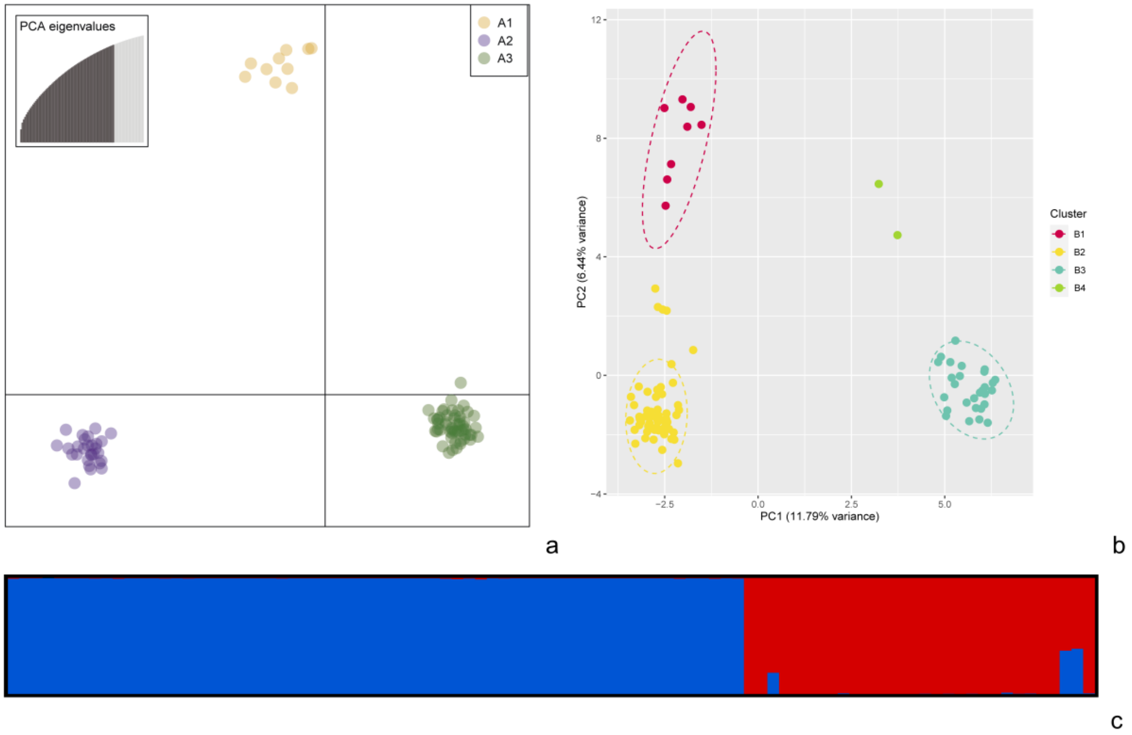

Supplementary Fig. 10 Graphical outputs of the three clustering analyses employed to evaluate the genetic structure for 93 genotypes of the panel using 662 dominant markers. (a) Plotting of the panel onto the first two linear discriminants from a discriminant analysis of principal components (DAPC). A division into three clusters (A1, A2 and A3) was considered. **(b)** Projection of the panel onto the first two principal components (PCs) from a principal component analysis (PCA). Genotypes are divided into four clusters (B1, B2, B3 and B4) as indicated by a k-means clustering analysis. Ellipses represent a 95% confidence interval. **(c)** Bayesian clustering performed on STRUCTURE. Each genotype is represented by a vertical bar split in accordance with the admixture coefficients for two clusters (C1 in dark blue and C2 in red). Genotypes are ordered according to ID number (see Supplementary Table 1)

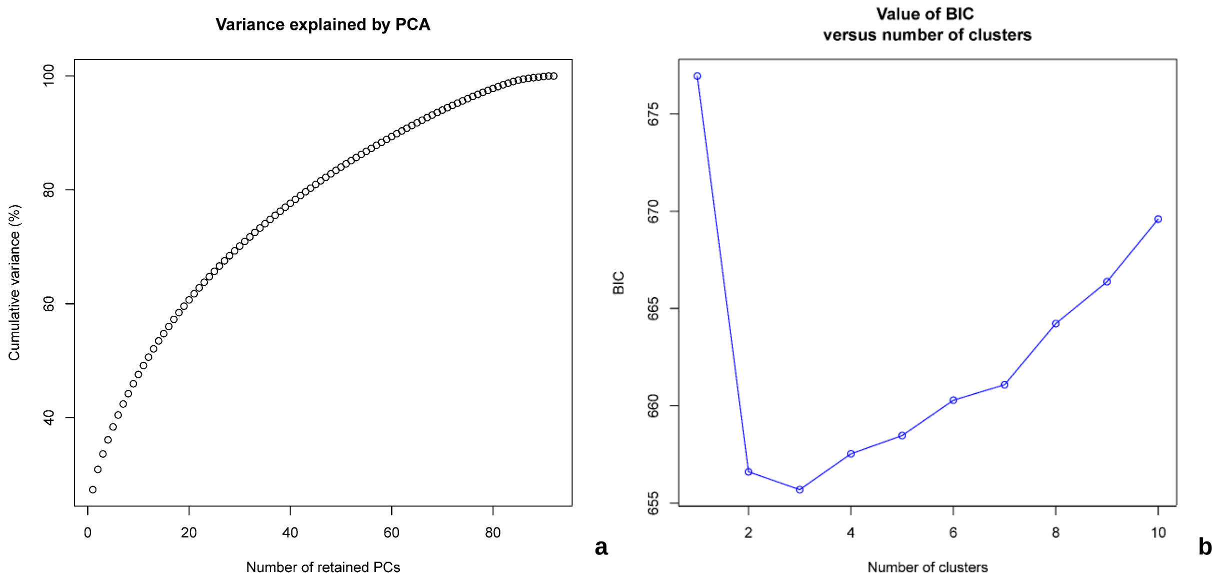

Supplementary Fig. 11 Graphical output of methods employed for the determination of the optimal number of genetic clusters in the panel based on a discriminant analysis of principal components (DAPC) using 70,888 codominant markers. (a) Cumulative variance explained by the principal components in the analysis. **(b)** Bayesian information criterion (BIC) versus number of clusters in k-means clustering

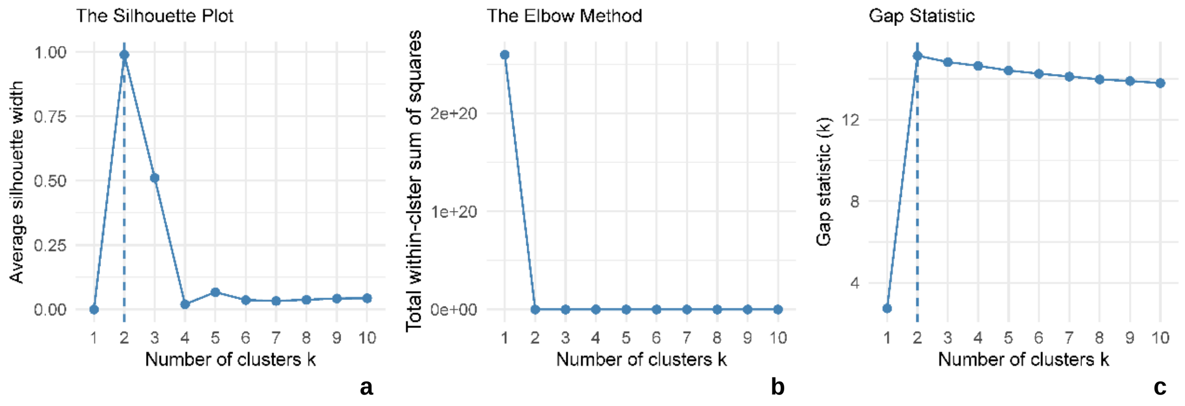

Supplementary Fig. 12 Graphical output of methods used for determining the optimal number of clusters in the panel from a principal component analysis (PCA) using 70,888 codominant markers. (a) Silhouette plot. **(b)** Elbow method. **(c)** Gap statistic

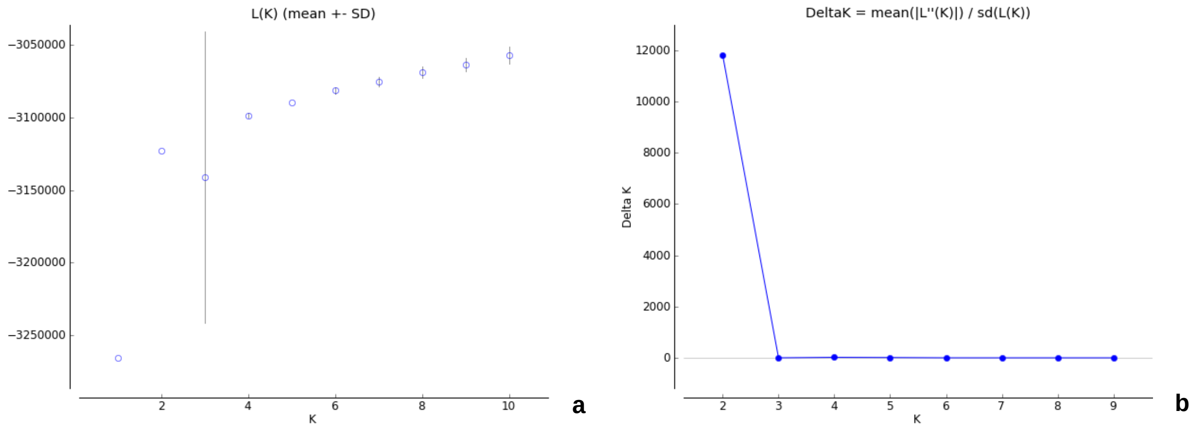

Supplementary Fig. 13 Graphical output of methods used for the determination of the optimal number of genetic clusters in the panel based on the STRUCTURE Bayesian clustering analysis using 7,000 codominant markers. (a) Mean LnP(D) for K = 1-10. **(b)** Mean ΔK for K = 1-10

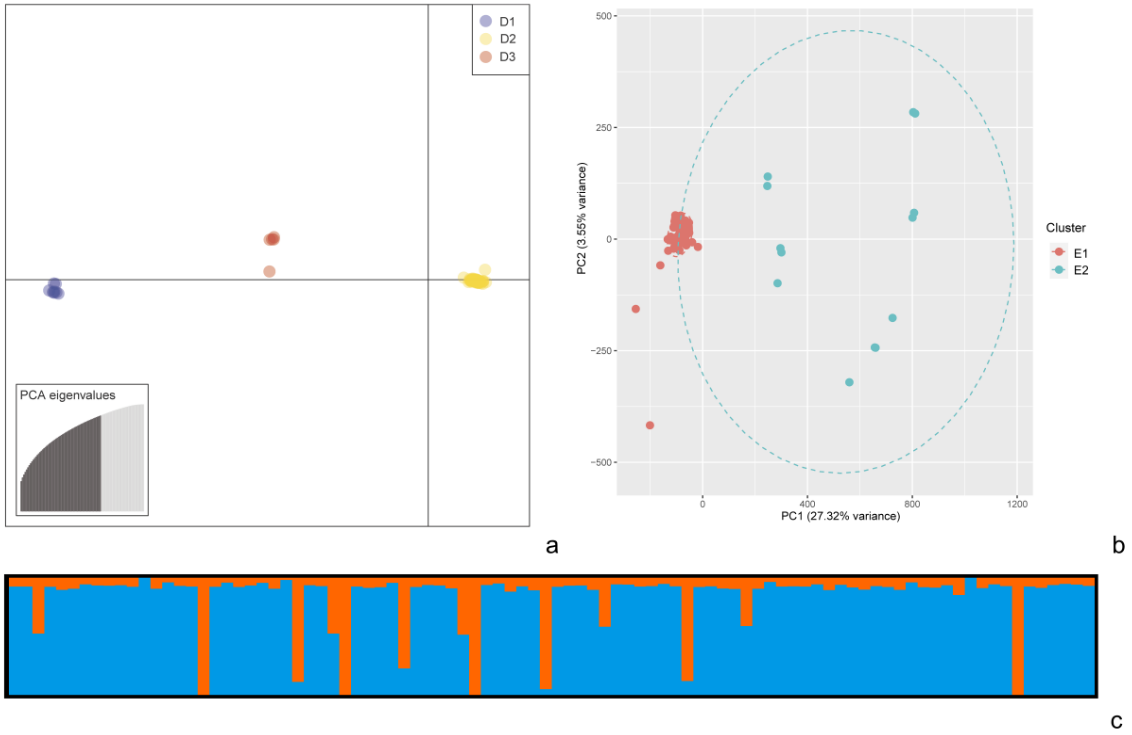

Supplementary Fig. 14 Graphical outputs of the three clustering analyses employed to evaluate the genetic structures for 92 genotypes in the panel using codominant markers. (a) Plotting of the panel onto the first two linear discriminants from a discriminant analysis of principal components (DAPC) using 70,888 single-nucleotide polymorphisms (SNPs) and insertions and deletions (indels). A division into three clusters (D1, D2 and D3) was considered. **(b)** Projection of the panel onto the first two principal components (PCs) from a principal component analysis (PCA) using 70,888 SNPs and indels. The genotypes were divided into two clusters (E1 and E2) as indicated by k-means clustering analysis. Ellipses represent a 95% confidence interval. **(c)** Bayesian clustering performed on STRUCTURE using 7,000 SNP and indel markers. Each genotype is represented by a vertical bar that is split in accordance with the admixture coefficients for two clusters (F1 in blue and F2 in orange). The genotypes are ordered according to ID number (see Supplementary Table 1)

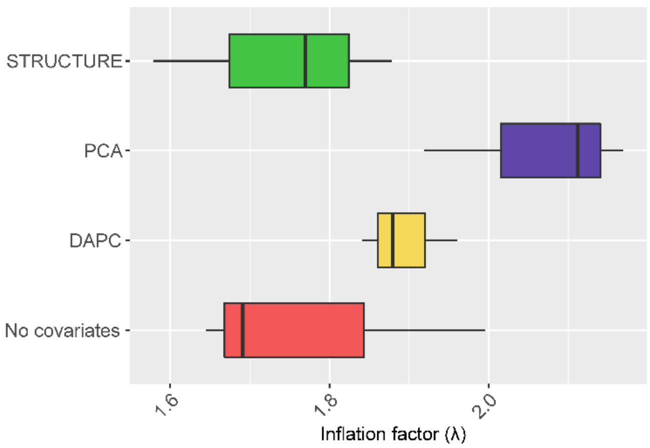

Supplementary Fig. 15 Genomic inflation factor (λ) of the fixed and random model circulating probability unification (FarmCPU) models for the three SCYLV resistance traits analyzed, including different population structure matrices as covariates. Abbreviations: DAPC, discriminant analysis of principal components; PCA, principal components analysis

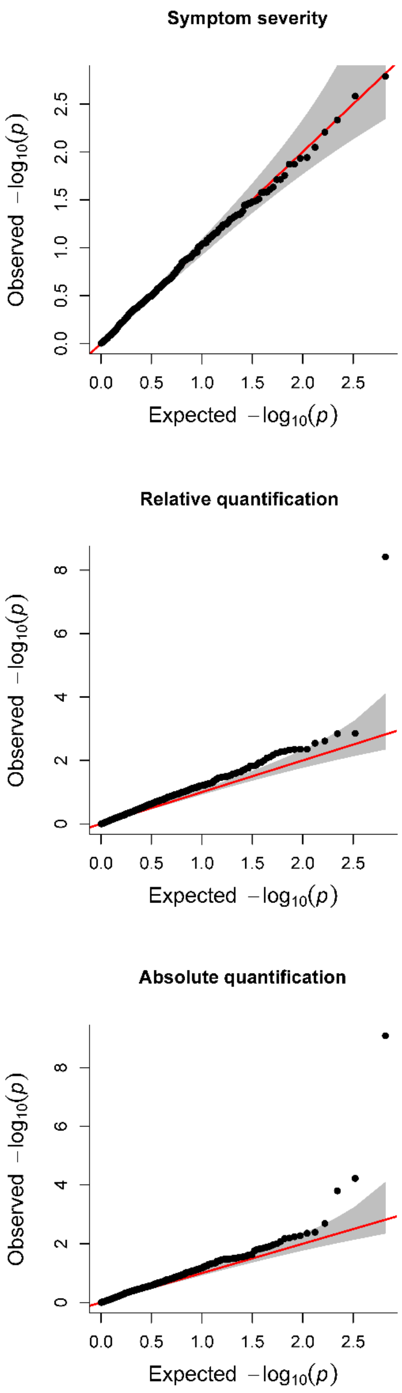

Supplementary Fig. 16 Quantile-quantile (Q-Q) plots generated in the fixed and random model circulating probability unification (FarmCPU) analyses using the best linear unbiased predictor (BLUP) values of each of the three analyzed traits and 622 dominant markers

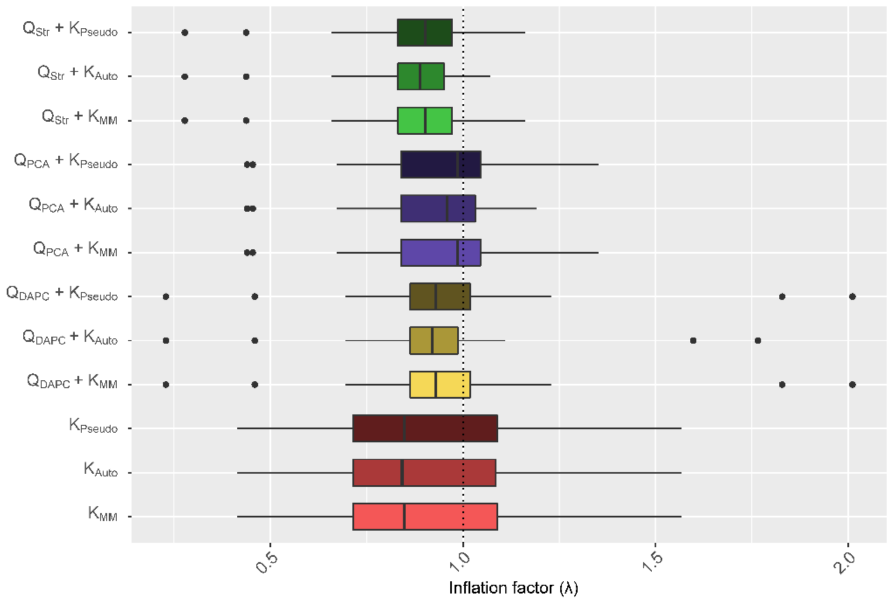

Supplementary Fig. 17 Genomic inflation factor (λ) of twelve association models for the three SCYLV resistance traits analyzed, including different population structure (Q) and kinship (K) matrices as covariates. Abbreviations: Auto, complete autopolyploid model; DAPC, discriminant analysis of principal components; MM, MM^T realized relationship model; PCA, principal components analysis; Pseudo, pseudodiploid model; Str, STRUCTURE Bayesian clustering

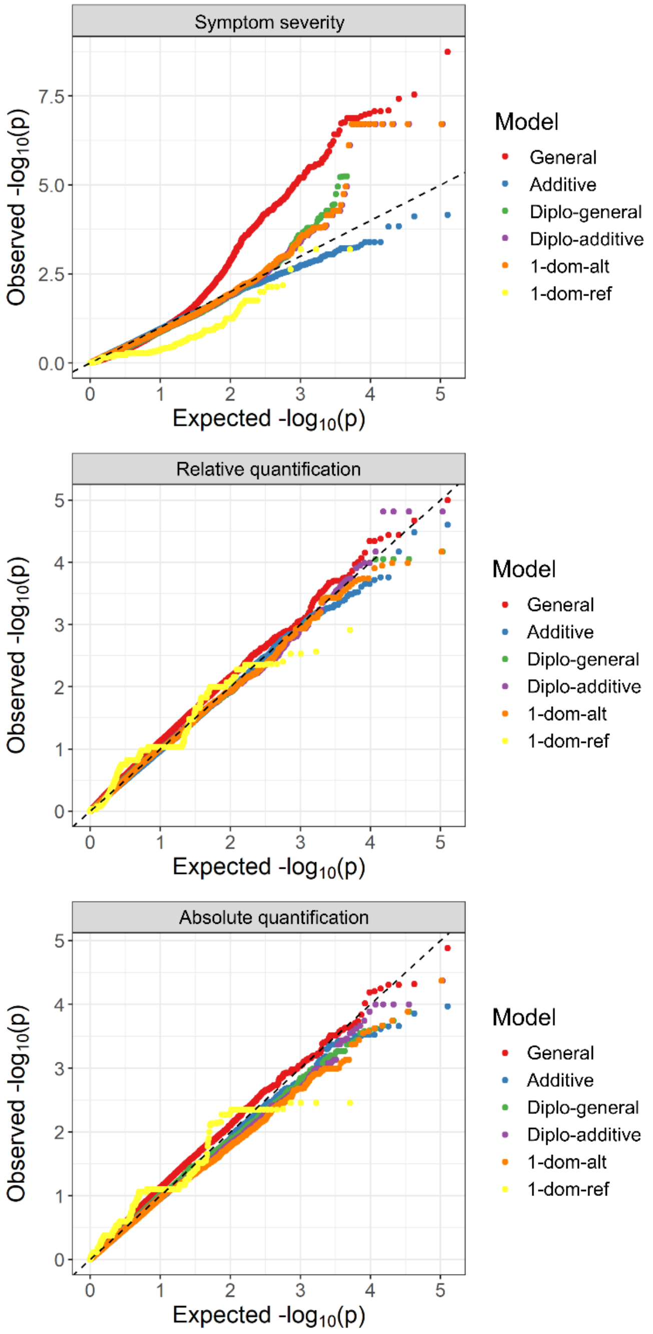

Supplementary Fig. 18 Q-Q plots generated in the association analysis using the best linear unbiased predictor (BLUP) values of the three SCYLV resistance traits analyzed. A realized relationship kinship matrix and the first three PCs from a PCA were used as covariates. Six different models were tested: general, additive, simplex dominant reference (1-dom-ref), simplex dominant alternative (1-dom-alt), diploidized general (diplo-general) and diploidized additive (diplo-additive)

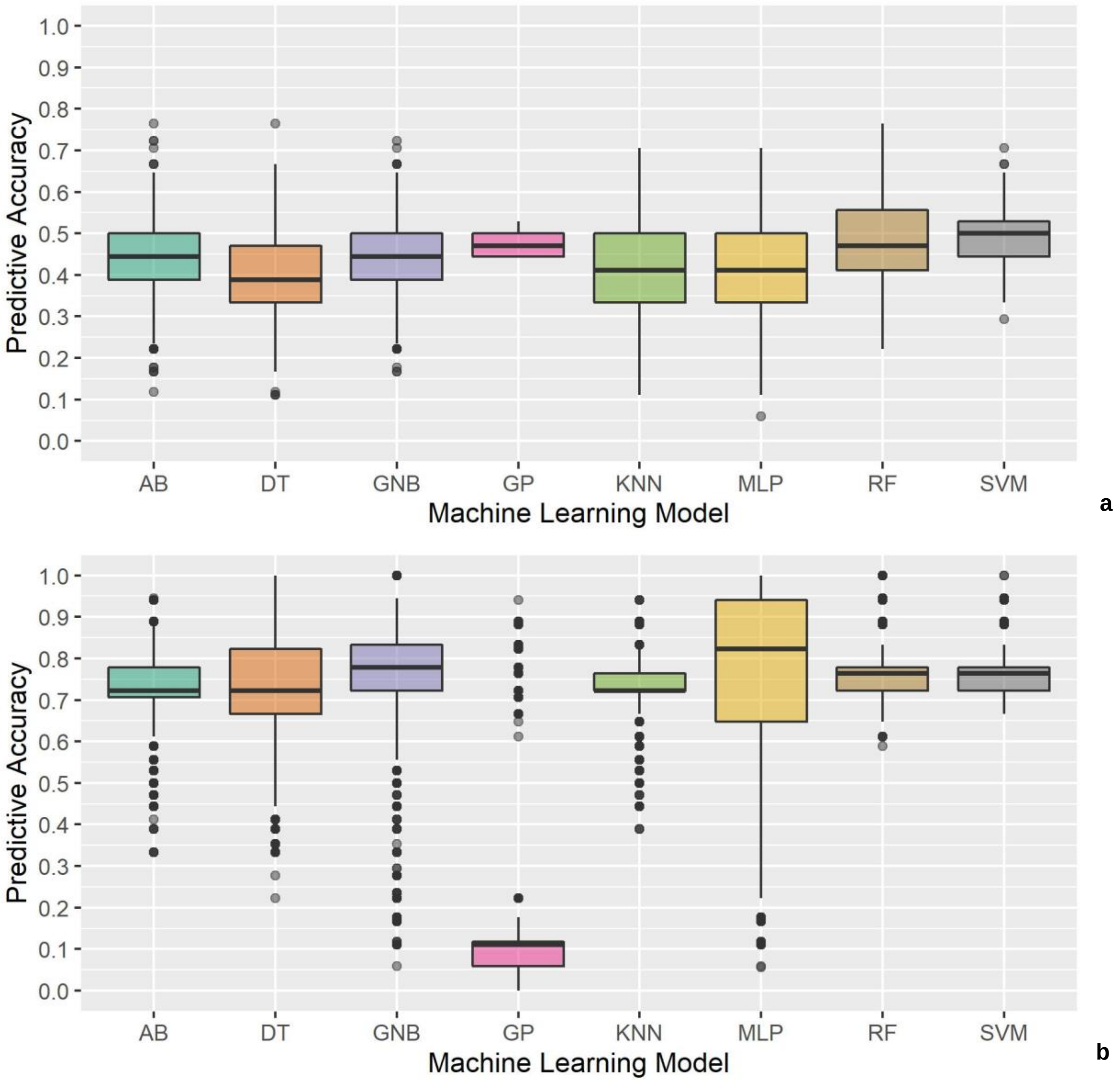

Supplementary Fig. 19 Distribution of predictive accuracies of each machine learning approach employed to predict clusters associated with SCYLV resistance. (a) Prediction of clustering by SCYLV titer determined by RT-qPCR (Q). **(b)** Prediction of clustering by SCYLV titer and SCYL symptom severity (SQ). The machine learning models tested were adaptive boosting (AB), decision tree (DT), Gaussian naive Bayes (GNB), Gaussian process (GP), K-nearest neighbor (KNN), multilayer perceptron neural network (MLP), random forest (RF) and support vector machine (SVM)

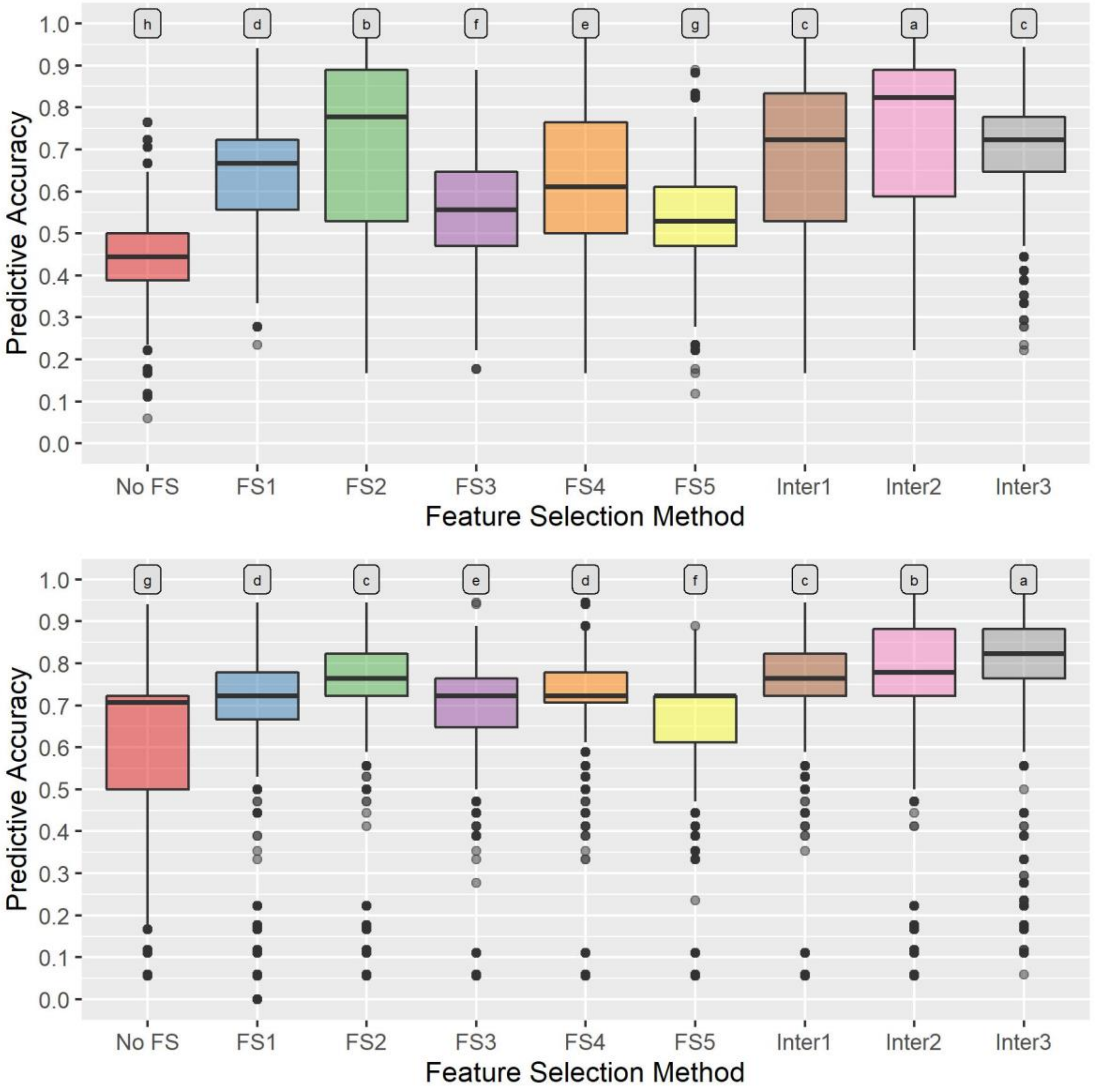

a

b

Supplementary Fig. 20 Distribution of predictive accuracies of all machine learning approaches employed to predict clusters associated with SCYLV resistance using each marker dataset obtained by feature selection (FS) strategies. (a) Prediction of clustering by SCYLV titer determined by RT-qPCR (Q). **(b)** Prediction of clustering by SCYLV titer and SCYL symptom severity (SQ). FS methods tested were gradient tree boosting (FS1), L1-based FS through a linear support vector classification system (FS2), extremely randomized trees (FS3), univariate FS using ANOVA (FS4) and RF (FS5), in addition to the intersection of markers selected by at least two of all FS methods (Inter1), the intersection of at least two of the three best FS methods (Inter2) and the intersection of the three best FS methods (Inter3). Letters above boxplots indicate significantly different groups identified by ANOVA followed by Tukey’s test
